# Structural characterization of *Linum usitatissimum* hydroxynitrile lyase: A new cyanohydrin decomposition mechanism involving a cyano-zinc complex

**DOI:** 10.1016/j.jbc.2022.101650

**Published:** 2022-01-29

**Authors:** Daijun Zheng, Makoto Nakabayashi, Yasuhisa Asano

**Affiliations:** 1 Biotechnology Research Center and Department of Biotechnology, Toyama Prefectural University, Imizu, Toyama, Japan; 2 Faculty of Pharmacy, Osaka Ohtani University, Tondabayashi, Osaka, Japan

**Keywords:** enzyme mechanism, enzyme structure, hydroxynitrile lyase, nicotinamide adenine dinucleotide (NAD), X-ray crystallography, zinc, ADH, alcohol dehydrogenase, BCN, (*R*)-2 -butanone cyanohydrin, CV, column volume, HNL, hydroxynitrile lyase, KPB, potassium phosphate buffer, *Lu*HNL, hydroxynitrile lyase from *Linum usitatissimum*

## Abstract

Hydroxynitrile lyase from *Linum usitatissimum* (*Lu*HNL) is an enzyme involved in the catabolism of cyanogenic glycosides to release hydrogen cyanide upon tissue damage. This enzyme strictly conserves the substrate- and NAD(H)-binding domains of Zn^2+^-containing alcohol dehydrogenase (ADH); however, there is no evidence suggesting that *Lu*HNL possesses ADH activity. Herein, we determined the ligand-free 3D structure of *Lu*HNL and its complex with acetone cyanohydrin and (*R*)-2-butanone cyanohydrin using X-ray crystallography. These structures reveal that an A-form NAD^+^ is tightly but not covalently bound to each subunit of *Lu*HNL. The restricted movement of the NAD+ molecule is due to the “sandwich structure” on the adenine moiety of NAD^+^. Moreover, the structures and mutagenesis analysis reveal a novel reaction mechanism for cyanohydrin decomposition involving the cyano-zinc complex and hydrogen-bonded interaction of the hydroxyl group of cyanohydrin with Glu323/Thr65 and H_2_O/Lys162 of *Lu*HNL. The deprotonated Lys162 and protonated Glu323 residues are presumably stabilized by a partially desolvated microenvironment. In summary, the substrate binding geometry of *Lu*HNL provides insights into the differences in activities of *Lu*HNL and ADH, and identifying this novel reaction mechanism is an important contribution to the study of hydroxynitrile lyases.

Hydroxynitrile lyases (HNLs) are primarily found in higher plants (1, 2), microorganisms (3, 4, 5), and millipedes (6, 7) as crucial enzymes participating in the process of cyanogenesis, in which it was identified to catalyze the decomposition of cyanohydrins to corresponding carbonyl compounds and toxic hydrogen cyanide (HCN) (8, 9, 10). The toxic HCN is released as a defense compound to protect from intruders. The HNLs identified to date can be classified into seven superfamilies that include FAD-binding oxidoreductase (*Pa*HNL (11, 12, 13, 14), *Pm*HNL (15), *Ps*HNL (16), *Ej*HNL (17, 18)), α/β-hydrolase fold (*At*HNL (19, 20), *Me*HNL (21, 22, 23, 24, 25, 26, 27, 28), *Hb*HNL (29, 30, 31, 32), *Sb*HNL (33, 34, 35), *Bm*HNL (36, 37)), dimeric α+β barrel (*Pe*HNL (38, 39)), lipocalin-like fold (*Chua*HNL (6, 40, 41), *Plam*HNL (42)), cupin (*Ac*HNL (4), *Psm*HNL (5), *Bp*HNL (5), *Gt*HNL (3)), bet-v1 like fold (*Dt*HNL (43)), and Zn^2+^-dependent alcohol dehydrogenase (*Lu*HNL (44, 45, 46, 47)). Among these, one or more HNL structures have been determined in each superfamily, except for the Zn^2+^-dependent alcohol dehydrogenase superfamily. In early 1981, it had demonstrated that the microsomal preparations from dark-grown *Linum usitatissimum* (*linen flax*) seedlings can transfer L-valine to acetone cyanohydrin, the precursor of the cyanogenic glucoside linamarin, which is the stock form of HCN *in vivo* (Fig. 1) (48). In 1987, *Lu*HNL, the only member of the Zn^2+^-dependent alcohol dehydrogenase superfamily, was purified for the first time from young seedings of flax (*L. usitatissimum* L.). It was characterized as a dimer with a subunit molecular mass of 42,000 Da (44). In 1997, the full-length cDNA encoding *Lu*HNL was isolated and cloned into *Escherichia coli*. The amino acid sequence of *Lu*HNL showed significant similarities to the alcohol dehydrogenase (ADH) family, rather than to other known HNLs. From the sequence alignment of *Lu*HNL and ADHs, it was found that the residues coordinating with Zn^2+^ ions and the ADP-binding βαβ unit motif in ADHs were highly conserved in *Lu*HNL. However, neither ADH activity in *Lu*HNL nor HNL activity in ADH was detected. From the loss of inhibition of *Lu*HNL activity with the addition of Zn^2+^ chelators, it was concluded that the Zn^2+^ ions were not directly involved in the catalysis of cyanohydrin cleavage (45). Furthermore, the subsequent site-directed mutagenesis analysis of *Lu*HNL by its overexpression in *Pichia pastoris* indicated that the residues involved in catalysis of Zn^2+^-ADHs were also functionally important in *Lu*HNL. From these results, it was presumed that all the Zn^2+^ ions in *Lu*HNL possess only the function of stabilizing the structure and not participating in the catalysis (46). All these conclusions seem reasonable based on the experimental results but not from direct evidence. Until now, no convincing evidence has been described to clarify these questions, and the catalytic mechanism of *Lu*HNL is also a mystery. To address these issues, the crystal structures of *Lu*HNL were determined using X-ray crystallography. In total, three structures of *Lu*HNL were determined: ligand-free *Lu*HNL (*Lu*HNL_lig_free, PDB ID: 7VB3), acetone cyanohydrin-complexed *Lu*HNL (*Lu*HNL_CNH, PDB ID: 7VB5), and (*R*)-2-butanone cyanohydrin-complexed *Lu*HNL (*Lu*HNL_BCN, PDB ID: 7VB6). Based on these crystal structures and site-directed mutagenesis analysis results, we proposed a catalytic mechanism for *Lu*HNL on cyanohydrin decomposition and elucidated the function of NAD^+^ and Zn^2+^ in *Lu*HNL. The structural information of *Lu*HNL also provided insights into the differences in its activity as compared with ADHs.

**Figure 1:**
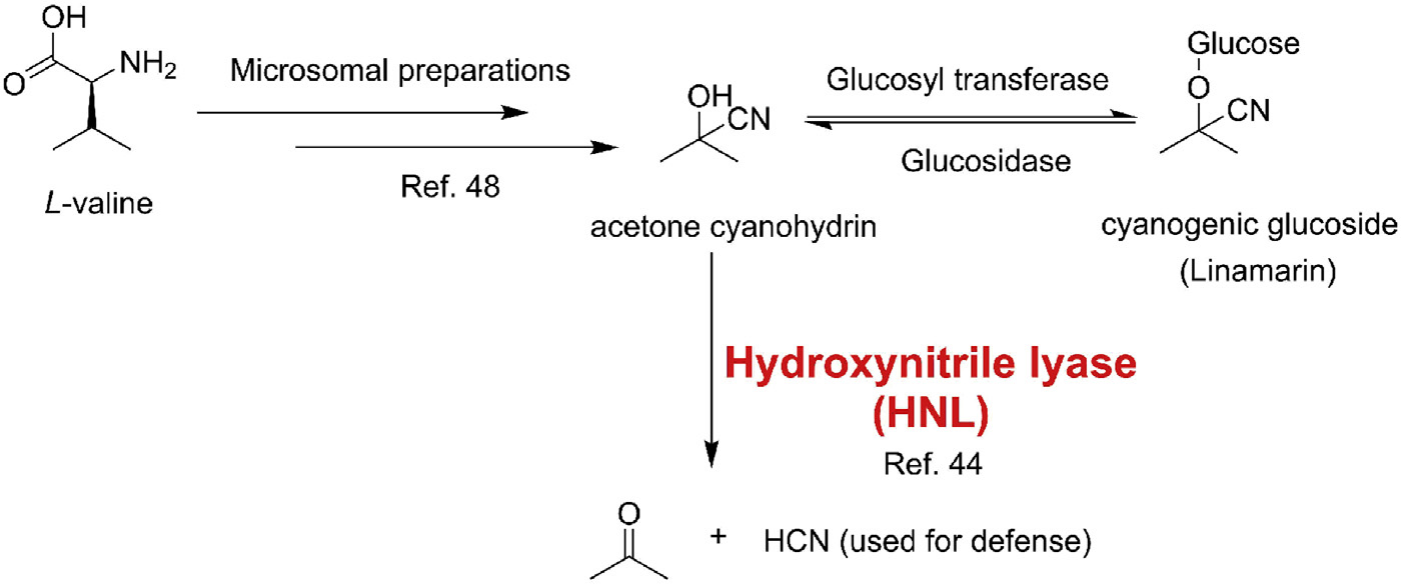
**The pathway for the conversion of L-valine to acetone and HCN in *Linum usitatissimum***.

## Results

### The overall structure of *Lu*HNL

**Figure 2:**
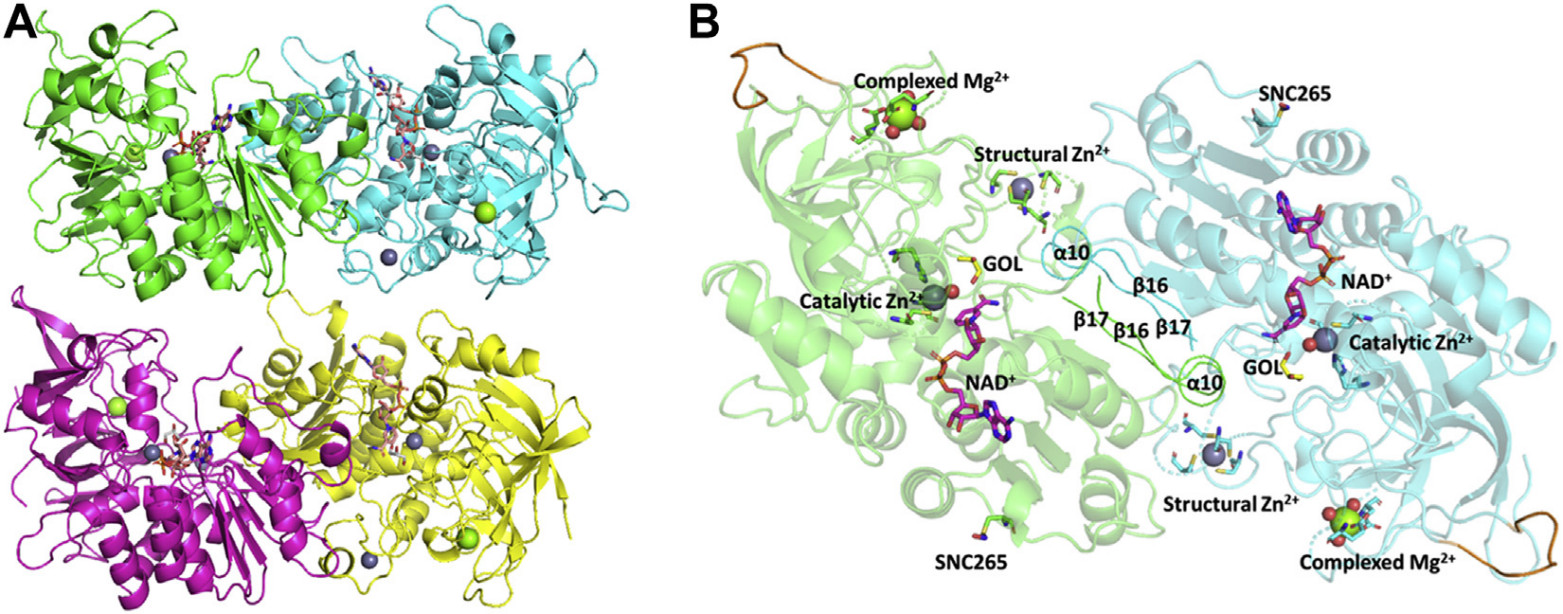
**Structure view of *Lu*HNL.***A*, the overall structure of *Lu*HNL with two parallel dimers observed per asymmetric unit. The four chains were colored in *green* (chain A), *cyan* (chain B), *magenta* (chain C), *yellow* (chain D), respectively. *B*, structural features observed in *Lu*HNL. As indicated in the picture, two Zn^2+^ ions (*gray spheres*), one NAD^+^ molecule, one Mg^2+^ ion (*green spheres*), and S-nitrosylation of Cys265 were identified in each chain. The water molecules complexed with metal ions were shown as *red spheres*. The GOL refers to glycerol molecule. The interface of two subunits (β16α10β17 moiety) was highlighted by *ribbon*. The parts with poor electron density map were marked as *gold color*. The protein structures were displayed using PyMOL (79).

**Figure 3:**
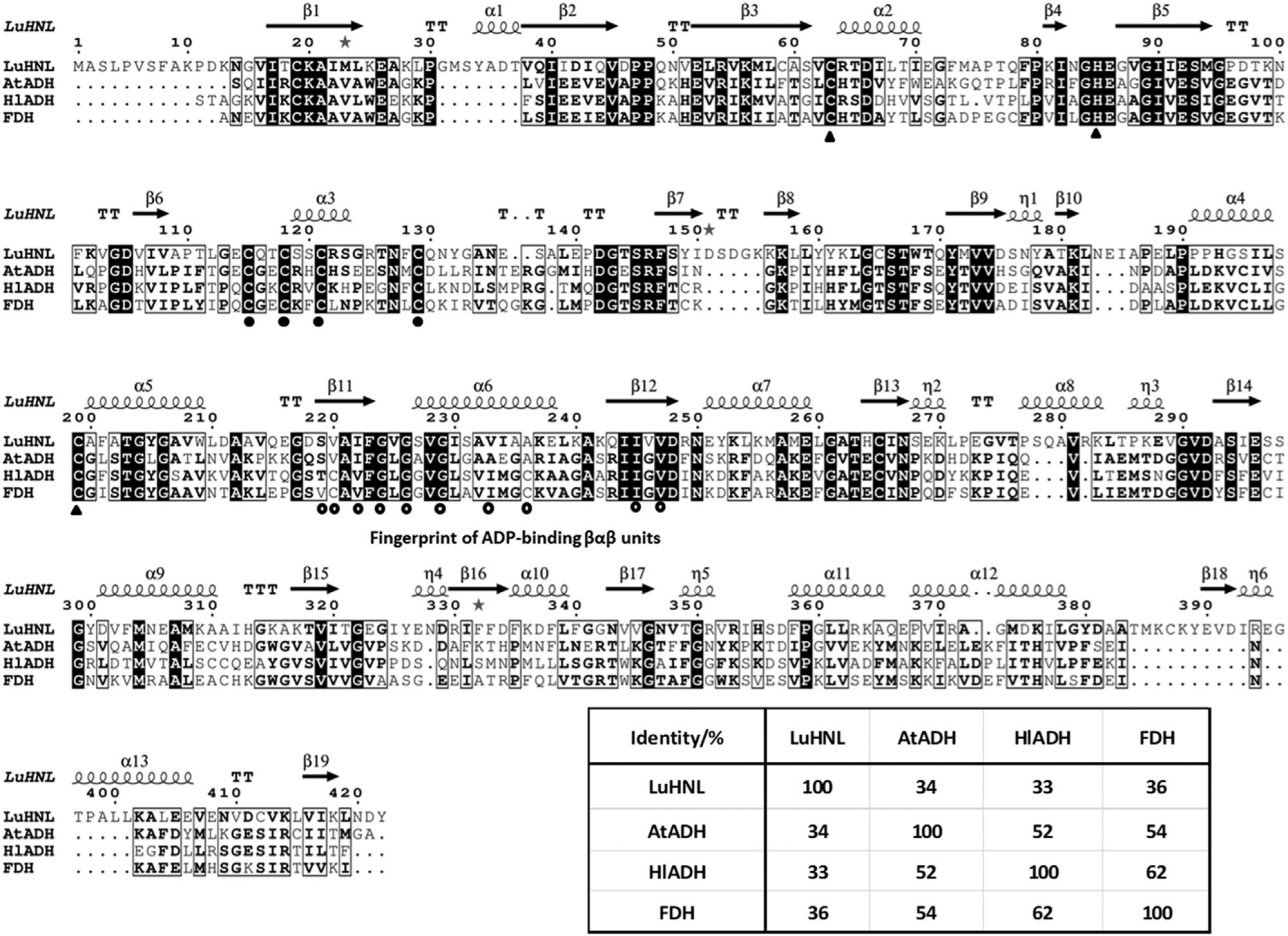
**Secondary structure–based multiple sequence alignment of *Lu*HNL with alcohol dehydrogenase (AtADH, PDB ID:****4RQU****, derived from *Arabidopsis thaliana*; Hl_ADH, PDB ID:****6ADH****, derived from *Equus caballus*)** (80, 81) **and formaldehyde dehydrogenase (FDH, PDB ID:****1M6H****, derived from *Homo sapiens*)** (82)**.** The secondary structural elements were shown as α-helices (*medium squiggles* with α symbols), 3_10_-helices (*small squiggles* with η symbols), β-strands (*arrows* with β symbols), strict β-turns (TT letters), and strict α-turns (TTT letters). The residues for catalytic Zn^2+^ coordination that are conserved in all four proteins were highlighted as *triangle symbols*. The four cysteines for structural Zn^2+^ coordination that are conserved in all four proteins were marked by *bold dots*. The fingerprint of ADH-binding βαβ units (53) were indicated as *circles*. The alignment was done using ESPript 3.0 server (http://espript.ibcp.fr/) (83).

Because of the low sequence identity of *Lu*HNL with structures of known proteins, the initial structure model of ligand-free *Lu*HNL (*Lu*HNL_lig_free) was built using the *BALBES* program (49). BALBES suggested a possible initial model of the human σ σ alcohol dehydrogenase (PDB ID: 1D1T, 32.47% sequence identity with *Lu*HNL) (50) and a subsequent molecular replacement was successfully processed. Further refinement was performed using *REFMAC5* (51) and *Phenix* (52). The crystal belongs to the monoclinic space group *P*2_1_ with unit-cell parameters *a* = 94.12 Å, *b* = 52.18, *c* = 168.51; *α* = 90.00, *β* = 95.01, *γ* = 90.00. The *R*_work_ and *R*_free_ values of the refined coordinate are 0.156 and 0.183 at 1.48 Å as shown in Table S1 (Supporting information, Section 1). Two dimers (dimer 1: chain A + B and dimer 2: chain C + D) in parallel as shown in Figure 2*A* were found in the asymmetric unit. The secondary structure of *Lu*HNL consists of 13 α-helices, 6 3_10_-helices, 19 β-sheets, 10 strict β-turns, and 1 strict α-turn, as shown in the Figure 3. The root-mean-square deviation (RMSD) between the Cα atoms of two dimers was 0.514 Å. Each of the two chains in dimer 1 (chain A + B) and dimer 2 (chain C + D) superimposed with an RMSD of 0.308 and 0.278 Å at the Cα atoms, respectively. Large conformational variations were observed in the β-turn between β7 and β8, β-turn between η2 and α8, and the residues around η4 and β16 of each chain in the dimers, which may be disordered as the electron density is not well defined. The conformational changes among four chains (Chain A, B, C, D) in terms of RMSD are 0.093 to 0.248 Å for β-turn between β7 and β8 (aa 146–158), 0.317 to 0.792 Å for β-turn between η2 and α8 (aa 268–283), and 0.092 to 0.327 Å for the residues around η4 and β16 (aa 326–334), respectively.

**Figure 4:**
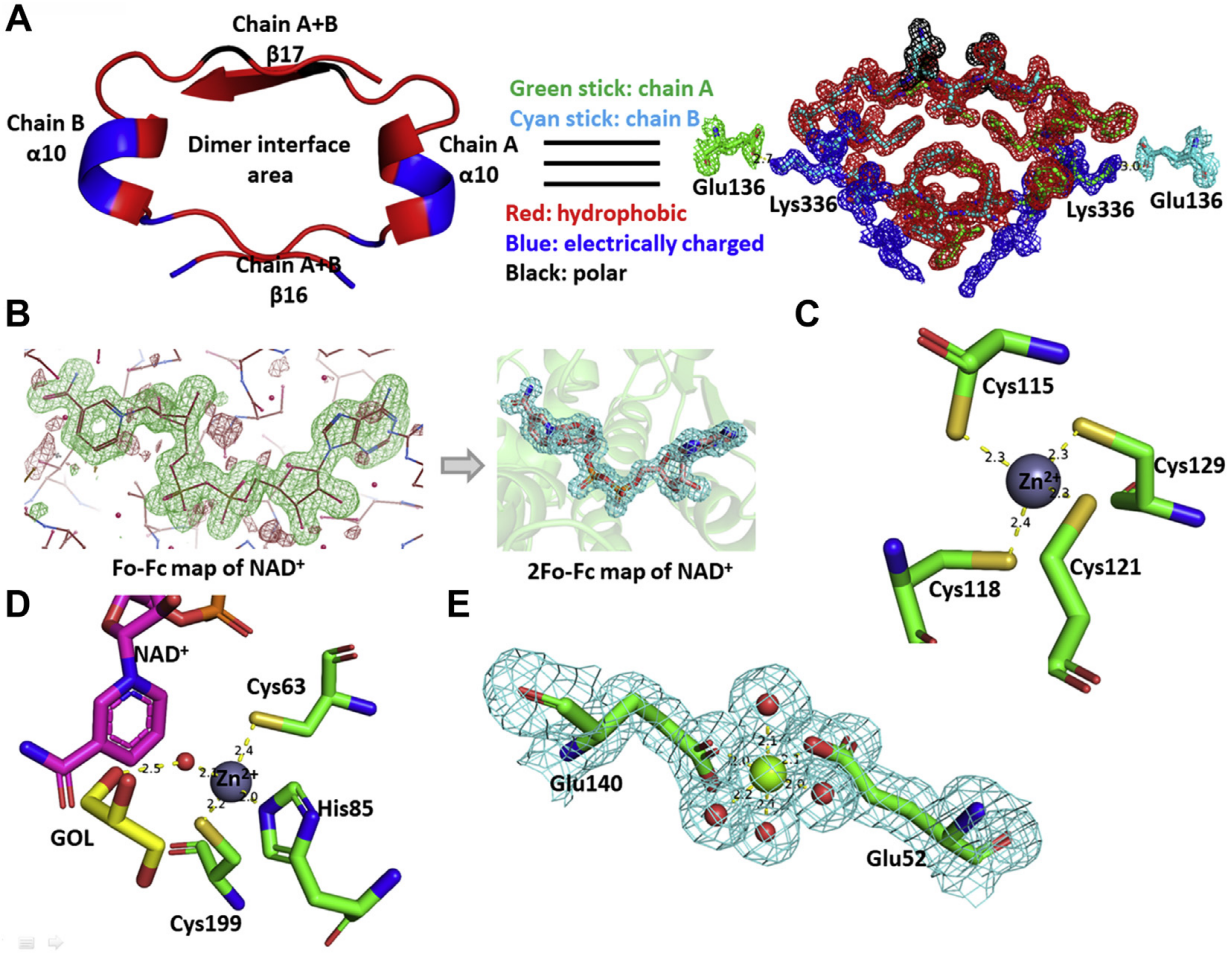
**Structural features of *Lu*HNL in detail.***A*, the interface area in a dimer; (*B*) the NAD^+^ molecule electron density of *F*_o_-*F*_c_ map (*green*) before inserting NAD^+^ and 2*F*_o_-*F*_c_ map after refinement; (*C*) the structural Zn^2+^ ion complexed with four cysteines; (*D*) the catalytic Zn^2+^ complexed with two cysteines, one histidine, and one water molecule (*red sphere*); (*E*) the complexed structure of Mg^2+^ coordinated with four water molecules (*red spheres*) and two glutamic acid residues. The GOL in *D* refers to glycerol. The *F*_o_-*F*_c_ map was displayed in COOT (76) and contoured at 3.0 σ. The positive omit map was displayed as *green*, and the negative omit map was displayed as *red*. The 2*F*_o_-*F*_c_ map was displayed using PyMOL (79) and contoured at 1 e^−^/Å^3^. The protein structures were displayed using PyMOL (79).

The interface region between two subunits in one dimer was in the β16α10β17 area (Fig. 2*B*), which contains 12 hydrophobic residues (Ile331, Phe332, Phe333, Phe335, Phe338, Leu339, Phe340, Gly341, Gly342, Val344, Val345, Gly346), four electrically charged residues (Arg330, Asp334, Lys336, Asp337), and one polar uncharged residue (Asn343). The central area of this βαβ unit is occupied by hydrophobic residues. The electrically charged residues and polar uncharged residue were located on the periphery and extended outward. A salt bridge between the ε-amino group in Lys336 and the carboxyl group in Glu136 of another chain was observed at 2.7 to 3.0 Å (Fig. 4*A*).

**Figure 5:**
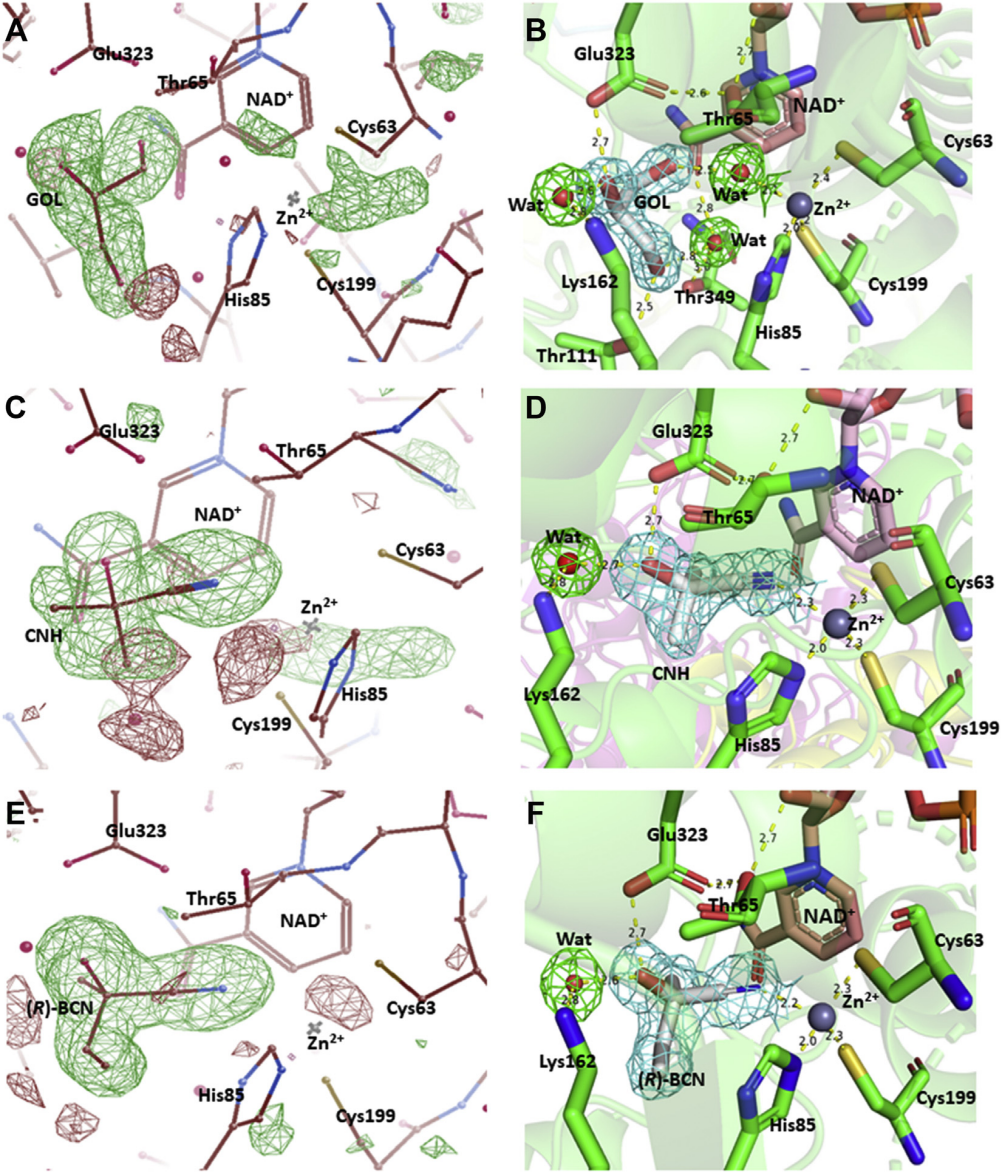
**Catalytic sites in *Lu*HNL.***A*, the *F*_o_-*F*_c_ omit map (*green*) of GOL in ligand-free structure of *Lu*HNL before inserting GOL; (*B*) the ligand-free structure of *Lu*HNL; (*C*) the *F*_o_-*F*_c_ omit map (*green*) of CNH in *Lu*HNL-CNH complex before inserting the CNH molecule; (*D*) the complex structure of *Lu*HNL with acetone cyanohydrin; (*E*) the *F*_o_-*F*_c_ omit map (*green*) of (*R*)-BCN in the *Lu*HNL–BCN complex before inserting the (*R*)-BCN molecule; (*F*) the complex structure of *Lu*HNL with (*R*)-2-butanone cyanohydrin. GOL refers to glycerol; CNH refers to acetone cyanohydrin; (*R*)-BCN refers to (*R*)-2-butanone cyanohydrin; Wat refers to water molecule. The *F*_o_-*F*_c_ map was displayed in COOT (75) and contoured at 3.0 σ. The positive omit map was displayed as *green*, and the negative omit map was displayed as *red*. The 2*F*_o_-*F*_c_ map was displayed using PyMOL (79) and contoured at 1 e^−^/Å^3^. The protein structures were displayed using PyMOL (79).

The electron density of NAD^+^ was observed in the crystal without the addition of exogenous NAD^+^ during enzyme crystallization or soaking of the crystal in an NAD^+^ solution before X-ray diffraction (Fig. 4*B*). The NAD^+^ molecule was bound to the β11α6β12 fold, a classic binding domain for ADP, which is consistent with the fingerprint for ADP binding that identified from the amino acid sequence of *Lu*HNL (Fig. 3) (53). Furthermore, two classical tetra-coordinated complexes of Zn^2+^ ions were observed. One was bonded to Cys115, Cys118, Cys121, and Cys 129 at a distance of 2.3 to 2.4 Å (Fig. 4*C*), and the second Zn^2+^ complex formed bonds with Cys63, His85, Cys199, and one molecule of water at a distance of 2.0 to 2.4 Å (Fig. 4*D*). Beyond the water molecule that bonded to the second Zn^2+^ ion, a glycerol molecule was trapped via hydrogen bond interaction with the water molecule and residues of Thr111, Lys162, Glu323, and Thr349 (Fig. 5, *A* and *B*), which implies that the second Zn^2+^ area is the catalytic site of *Lu*HNL. In addition, a hexa-coordinated complex of Mg^2+^ ion was observed on the enzyme surface that bonded with residues of Glu52, Glu140, and four molecules of water at 2.0 to 2.2 Å (Fig. 4*E*). The quantitative measurement of metal ions in *Lu*HNL using inductively coupled plasma mass spectrometry indicated that the contents of metal ions were 2.22 Zn^2+^ and 0.35 Mg^2+^ in each subunit, respectively (Table S2, Supporting information, Section 2). Moreover, it was noted that a positive *F*_o_-*F*_c_ omit map was attached to the thiol group of Cys265 in β13 during structure refinement, which suggests that the Cys265 was modified in *Lu*HNL. According to the possible modification forms of cysteine in protein (54), an S-nitrosylation form of Cys265 was proposed in *Lu*HNL, which fits the observed electron density. However, we failed to detect the S-nitrosyl group in *Lu*HNL solution by Saville’s method (55). Of interest, the S-nitrosyl group can be detected in lyophilized *Lu*HNL. The results suggest that the S-nitrosylation of Cys265 may be formed during crystallization or X-ray diffraction, rather than the posttranslational modification of *Lu*HNL.

### The complex structures of *Lu*HNL

**Figure 6:**
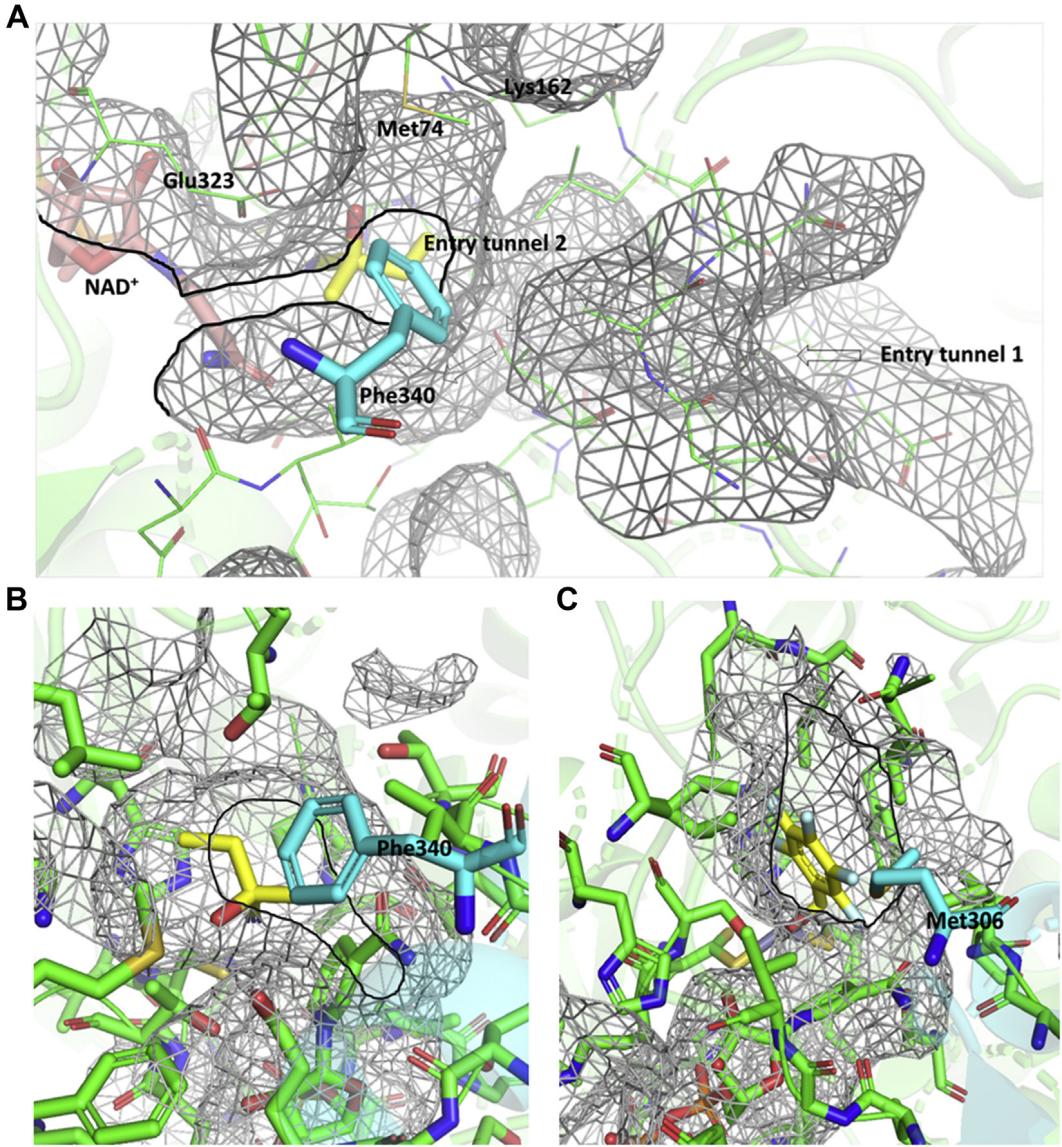
**Substrate entry tunnels in *Lu*HNL and Hl_ADH.***A*, substrate entry tunnels in *Lu*HNL_BCN; (*B*) substrate entry tunnel 2 in *Lu*HNL_BCN; (*C*) substrate entry tunnel in Hl_ADH (PDB ID: 4NFH) (60). Subunit A was displayed as *green*, and subunit B was displayed as *cyan*. The entry tunnel 1 in *Lu*HNL was indicated by *arrow*. The entry tunnel 2 of *Lu*HNL and the entry tunnel of Hl_ADH were marked by *bold black line*. The ligands in (*A*) and (*B*) were displayed as *yellow color*, which refer to 2-butanone cyanohydrin. The ligand in (C) was displayed as *yellow color*, which refers to 2,3,4,5,6-pentafluorobenzyl alcohol. The protein structures were displayed using PyMOL (79).

The structures for acetone cyanohydrin (CNH) complexed *Lu*HNL (*Lu*HNL_CNH) and (*R*)-2-butanone cyanohydrin (BCN) complexed *Lu*HNL (*Lu*HNL_BCN) were determined at a resolution of 1.58 and 1.72 Å, respectively. These two complex structures have similar unit cell dimensions with *Lu*HNL_lig_free, as summarized in Table S1 (Supporting information, Section 1). The RMSD between *Lu*HNL_lig_free and *Lu*HNL_CNH, *Lu*HNL_lig_free and *Lu*HNL_BCN was 0.524 and 0.309 Å, respectively, at Cα atoms of all four subunits. This indicates that the complex structures of *Lu*HNL did not change significantly as compared with the ligand-free *Lu*HNL structure. Large conformational variations were observed in the N-terminal, β-turns between β7 and β8, and β-turns between η2 and α8. The electron density of all the structural features that was observed in the *Lu*HNL_lig_free structure was also well defined in the complex structures, such as tightly bound NAD^+^ molecule, two tetra-coordinated Zn^2+^ ions, one hexa-coordinated Mg^2+^ ion, and S-nitrosylation of Cys265 in each subunit. In addition, the RMSD between *Lu*HNL_CNH and *Lu*HNL_BCN was 0.297 Å for the Cα atoms of all four chains. In the *Lu*HNL_CNH structure, the electron density of the acetone cyanohydrin (CNH) in the catalytic pocket was only observed in subunits of A, B, and C. However, the electron density of (*R*)-2-butanone cyanohydrin in the catalytic pocket of the *Lu*HNL_BCN structure was well defined in all four subunits. These two complexes indicate the same substrate binding pattern in the catalytic site (Fig. 5, *C*–*F*), which is different from the glycerol binding pattern in *Lu*HNL_lig_free as described above (Fig. 5*B*). In the substrate complexed structures, the nitrile group of the substrate replaced the water molecule observed in the *Lu*HNL_lig_free structure (Fig. 5*B*) to bond with the catalytic zinc ion coordinated with Cys63, His85, and Cys199 at a distance of 2.2 to 2.3 Å. The hydroxyl group of cyanohydrins oriented to form direct hydrogen bond interaction with Glu323 at a distance of 2.7 Å and indirect interaction with Lys162 *via* one molecule of water at a distance of 2.6 to 2.8 Å for each hydrogen bond. Furthermore, the hydrogen bond relay was extended to the O2D of NAD^+^ from Glu323 via Thr65 residue at 2.7 Å for each hydrogen bond. In addition, two substrate entry tunnels were observed in the subunit of *Lu*HNL, as shown in Figure 6*A*. The substrate entry tunnel 1 is a long and tortuous channel that connects the surface of the protein with the bottom of the catalytic pocket. Oppositely, the substrate entry tunnel 2 is located at the upper part of the catalytic pocket and close to the interface of the dimer (β16α10β17 fold). However, in the dimer structure of *Lu*HNL, the substrate entry tunnel 2 was completely shielded by the Phe340 on the helix α10 fragment of another subunit (Fig. 6*A*), resulting in a closed upper part of the catalytic pocket.

### The framework of NAD^+^-binding cavity

**Figure 7:**
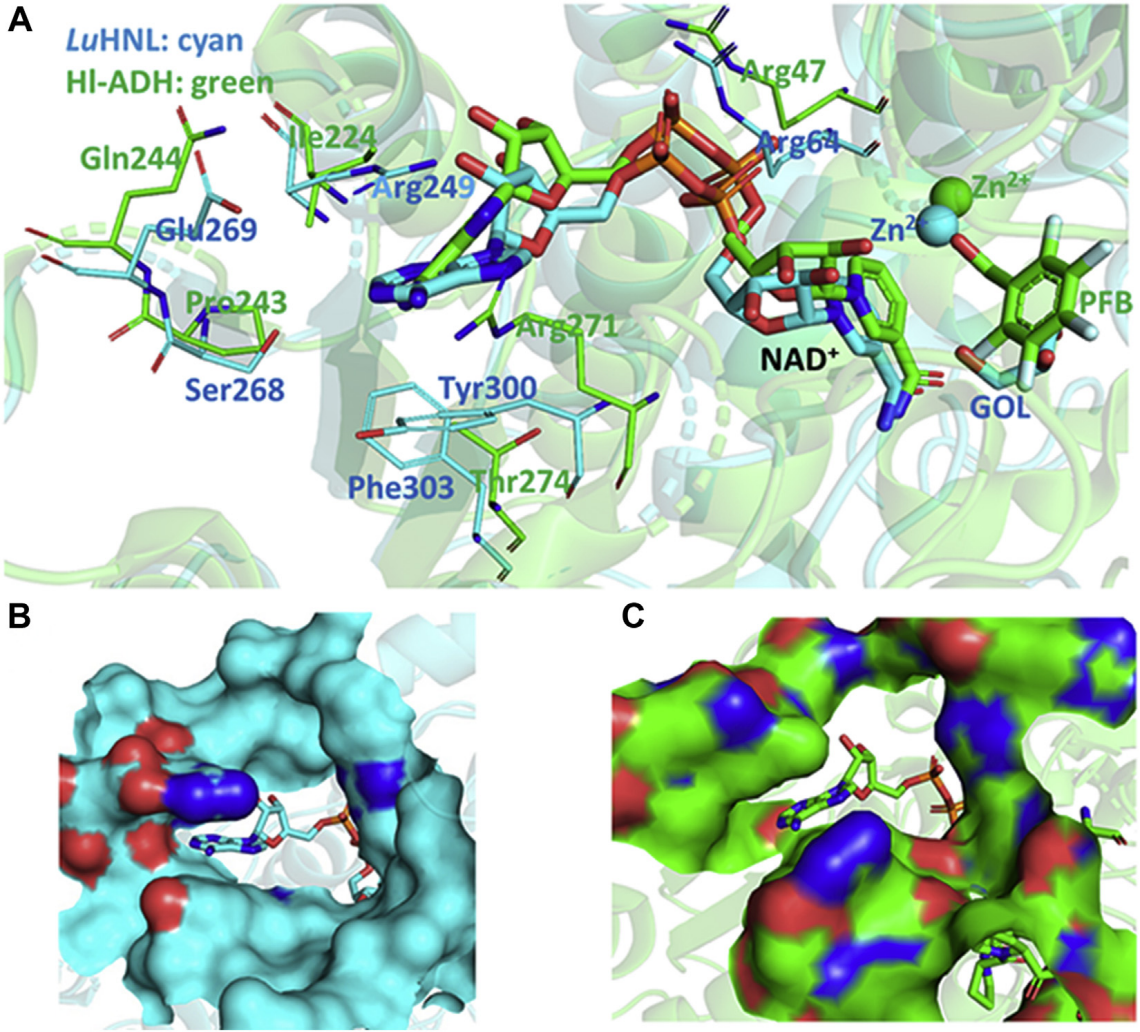
**Comparison of NAD**^**+**^**-binding cavities in *Lu*HNL and Hl_ADH.***A*, the superposition of NAD^+^-binding cavities of *Lu*HNL-lig-free and Hl_ADH (PDB ID: 4NFH) (60). *B*, the protein surface at the entrance of the NAD^+^-binding cavity in *Lu*HNL-lig-free structure. *C*, the protein surface at the entrance of the NAD^+^-binding cavity in Hl_ADH structure. GOL refers to glycerol, and PFB refers to 2,3,4,5,6-pentafluorobenzyl alcohol. The *Lu*HNL-lig-free structure was colored as cyan, and Hl_ADH structure was colored as green. The protein structures were displayed using PyMOL (79).

The well-defined electron density of NAD^+^ (Fig. 4*B*) in *Lu*HNL indicates that the cofactor NAD^+^ is tightly bound to the enzyme. Although several rare covalent bonding examples of NAD(P)^+^ with cysteine residue in enzyme were reported in aldehyde dehydrogenases (56, 57, 58), the release of NAD^+^ by denaturing the enzyme using 4 M guanidine suggests that NAD^+^ is trapped in the cavity via hydrogen bonding interactions rather than covalent bonding. The quantitative measurement of NAD^+^ showed that one subunit of *Lu*HNL contains approximately 0.68 molecule of NAD^+^ (Fig. S1, Supporting information, Section 3). From the anticonformation of NAD^+^, it was noted that it is the *re*-face of nicotinamide in NAD^+^ that approaches the catalytic sites, suggesting that the NAD^+^ in *Lu*HNL belongs to the A-form (59). Considering the diversity of ADHs and simplifying the description, we focus on the comparison of *Lu*HNL and well-studied horse liver ADH (Hl_ADH) in the following narrative. The superposition of the *Lu*HNL_lig_free structure with horse liver ADH (Hl_ADH, PDB ID: 4NFH) (60) showed a significant difference in the residues located at the entrance of the NAD^+^-bound cavity. In *Lu*HNL, the adenine part is buried in a “sandwich structure” of Arg249/adenine of NAD^+^/Tyr300, resulting in a twisted NAD^+^ molecule in the cavity (Fig. 7*A*). In such a situation, the twisted NAD^+^ molecule becomes less flexible, which may restrain its free movement. Moreover, the comparison of the protein surface in the entrance of NAD^+^-binding cavities of *Lu*HNL (Fig. 7*B*) and Hl_ADH (Fig. 7*C*) give us a more intuitive vision on the buried NAD^+^ of *Lu*HNL, in which the adenine part of NAD^+^ is embedded in a narrow space, rather than an open space as shown in Hl_ADH.

### The role of NAD^+^ in *Lu*HNL

To investigate the function of NAD^+^ in *Lu*HNL, we tried to prepare the apo-*Lu*HNL by refolding the denatured protein or to synthesize the apoenzyme by cell-free protein synthesis systems. However, both failed to make an apo-*Lu*HNL. Then a mutant of *Lu*HNL-R249G/S268A/E269L that mutated on the residues located at the entrance of NAD^+^-bound cavity was constructed and successfully expressed. In the purification of the enzyme using ion-exchange column of Mono Q 5/50 GL, three proteins (*Lu*HNL-S3, S4, and S5 in Fig. S2, Supporting information, Section 4) possessing the same size in SDS-PAGE were eluted out at different ionic strengths. The LC-MS/MS analysis revealed that all the proteins were *Lu*HNLs (data not shown). The CD-spectral analysis showed that the secondary structure of the first to be eluted enzyme (*Lu*HNL-S3) was different from that of the latter two (*Lu*HNL-S4, S5). The latter two enzymes (*Lu*HNL-S4, S5) had secondary structures similar to that of the *Lu*HNL wild type (*Lu*HNL-S2). However, except for the protein that was eluted last (*Lu*HNL-S5), the enzymes (*Lu*HNL-S3, S4) showed no activity. The specific activity of the last enzyme (*Lu*HNL-S5) on acetone cyanohydrin decomposition is 11.5 U/mg, about 23% of the wild-type enzyme activity (*Lu*HNL-S2, 49.8 U/mg). Furthermore, 0.35 molecule of NAD^+^/monomer was detected in the last protein (*Lu*HNL-S5), about 56% of the NAD^+^ content that was detected in the wild type (*Lu*HNL-S2, 0.62 molecule NAD^+^/monomer), whereas the other proteins (*Lu*HNL-S3, S4) lost NAD^+^ completely. We also found that the enzyme activity (*Lu*HNL-S1, S3, S4) could not be recovered by the addition of external NAD^+^, and no improvement in activity was observed for the active fractions of the *Lu*HNL-R249G/S268A/E269L (*Lu*HNL-S5, 12 U/mg) or *Lu*HNL-wild (*Lu*HNL-S2, 48.8 U/mg) in the presence of NAD^+^ (Fig. S2, Supporting information, Section 4).

### Site-directed mutagenesis analysis of *Lu*HNL

**Figure 8:**
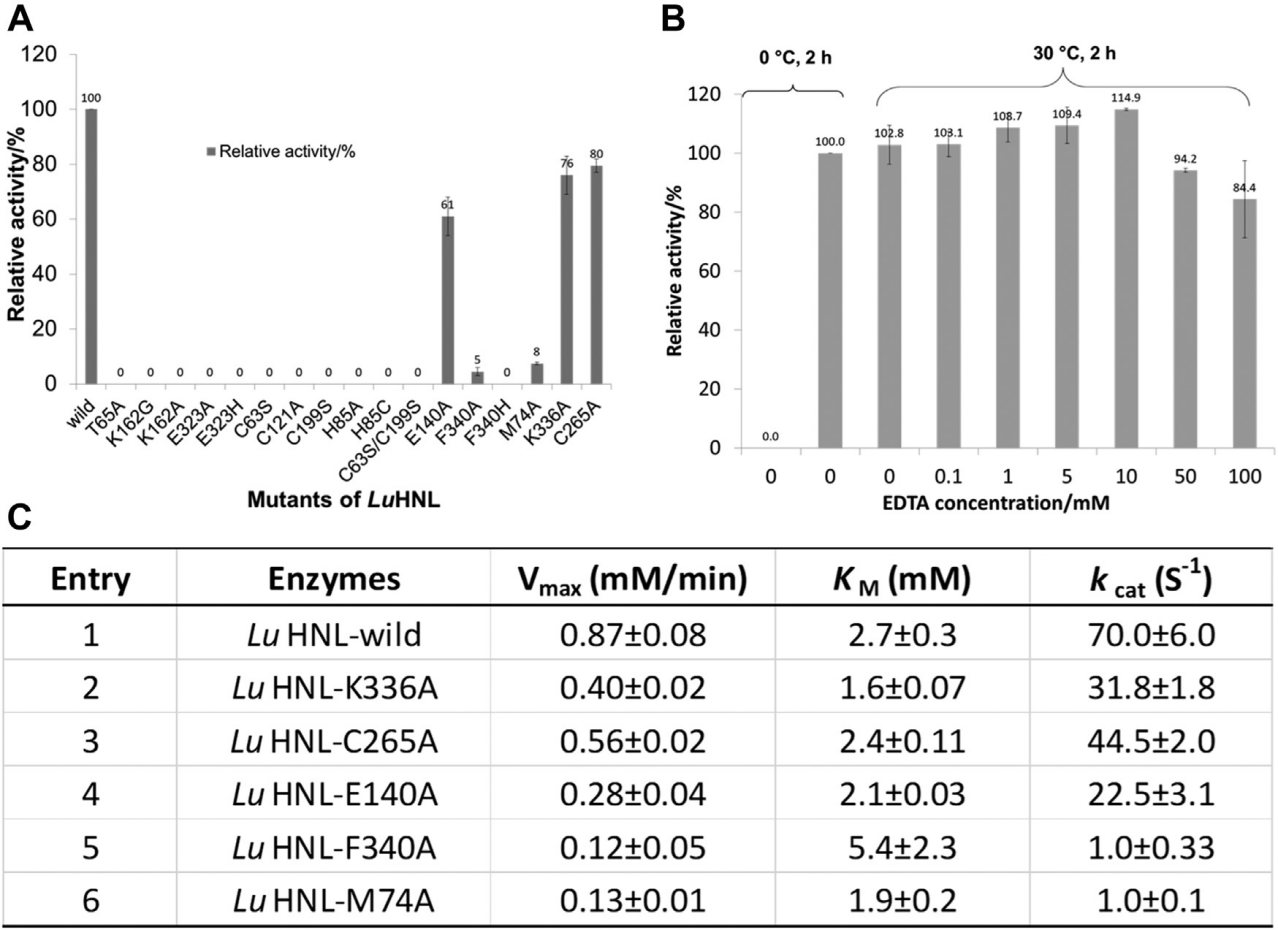
**Mutagenesis analysis and EDTA effect investigation of *Lu*HNL.***A*, site-directed mutagenesis analysis of *Lu*HNL. The activity of *Lu*HNL-wild type was set at 100%. *B*, the EDTA effect on *Lu*HNL activity. The *Lu*HNL enzymes were incubated with a range of concentration of EDTA (0–100 mM) at 30 °C for 2 h. The activity of *Lu*HNL incubated at 0 °C for 2 h was set at 100%. *C*, the kinetic parameters for active mutants of *Lu*HNL (The details are shown in Supporting information, Section 6).

Site-directed mutagenesis of the catalytic residues was carried out according to the substrate binding geometry in *Lu*HNL. Activity measurements on the acetone cyanohydrin decomposition revealed that the mutants of T65A, K162G, K162A, E323A, E323H, C63S, C199S, H85A, H85C, and C63S/C199S completely lost activity as compared with the wild type (Fig. 8*A*). In addition, the mutation of Cys121 to alanine also inactivated the enzyme completely, which is consistent with the findings of a previous report (46). Moreover, alanine mutation analysis of the residues that coordinated with Mg^2+^ was also performed. The E52A was expressed in an insoluble form using *E. coli* as the host, but E140A was successfully expressed and purified. The activity analysis suggests that the mutant of E140A possesses approximately 61% activity compared with the wild type (Fig. 8*A*, details are shown in Supporting information, Section 5). The mutation of Cys265 to alanine also showed a negligible effect on enzyme activity, and approximately 80% residual activity was detected for C265A (Fig. 8*A*, details are shown in Supporting information, Section 5). Subsequently, the mutation analysis on the residues of F340, K336 that are located at the interaction area (on the helix α10) of two subunits was also carried out. Compared with the wild type, the F340A showed 5% residual activity, whereas the F340H lost activity completely (Fig. 8*A*, details are shown in Supporting information, Section 5). The mutant of *Lu*HNL-K336A did not show significant change in enzyme activity, and approximately 76% residual activity was detected (Fig. 8*A*, details are shown in Supporting information, Section 5). Of note, when Met74, a residue located at the top of the catalytic pocket (Fig. 6*A*), was mutated to alanine, it resulted in a significant decrease in enzyme activity, and only 8% residual activity was detected (Fig. 8*A*, details are shown in Supporting information, Section 5). Then the kinetic parameters of *Lu*HNL-wild and its active mutants were determined (details are shown in Supporting information, Section 6). As shown in Figure 8*C*, the *K*_M_ value of the mutants is similar to that of the wild-type enzyme (*K*_M_ = 2.7 ± 0.3 mM) except for the mutant of *Lu*HNL-F340A (*K*_M_ = 5.4 ± 2.3 mM). It means the mutation of Phe340 to Ala340 decreases the affinity of the enzyme to the substrate.

### Effects of EDTA on *Lu*HNL activity

In a previous study (45), it was observed that the *Lu*HNL activity cannot be inhibited by addition of *o*-phenanthroline (dissociation constant of monophenanthroline-zinc: *K*_d_ = 3.7 × 10^−7^ M (61)), a competitive inhibitor of horse liver alcohol dehydrogenase (Hl_ADH) chelating with Zn^2+^ ion in the enzyme (62). The findings suggested that Zn^2+^ ions in *Lu*HNL are not directly involved in cyanohydrin decomposition (45). Here, we tested the effect of a much stronger Zn^2+^ chelator (dissociation constant of EDTA-zinc: *K*_d_ = 2.3 × 10^−14^ M (63)) on the activity of *Lu*HNL by incubation of the enzyme with 0 to 100 mM EDTA at 30 °C for 2 h (details are shown in Supporting information, Section 7), as a previous study did on yeast alcohol dehydrogenase (YADH), in which 64% of Zn^2+^ ions were removed by incubating the YADH with 100 mM EDTA at 30 °C for 2 h, resulting in a strong inhibitory effect of EDTA on YADH activity (64). However, the results showed that approximately 84% residual activity was detected on acetone cyanohydrin decomposition after incubation of *Lu*HNL with 100 mM EDTA at 30 °C for 2 h (Fig. 8*B*). And the metal content analysis showed that there is still 68% residual Zn^2+^ in the *Lu*HNL after incubation with 100 mM EDTA for 2 h. It suggests that the low extracting efficiency of EDTA on the Zn^2+^ from *Lu*HNL is the reason for the slight inhibitory effect of EDTA on *Lu*HNL activity. Compared with the EDTA effect on YADH, it implies that the Zn^2+^ ion is bound tightly in *Lu*HNL and important for *Lu*HNL activity. The need of Zn^2+^ ion for *Lu*HNL activity is also supported by the results that the enzyme activity was improved 1.03- to 1.20-fold by addition of 0.1 to 50 mM of Zn^2+^ in the activity assay mixture.

## Discussion

*Lu*HNL, a hydroxynitrile lyase that independently evolved from an ancestor protein possessing an ADP-binding βαβ domain, has unique structural features that differ from those of all known hydroxynitrile lyases. The sequence alignment of *Lu*HNL suggests that its structure is closer to that of Zn^2+^-containing alcohol dehydrogenase (ADH), sharing the substrate and NAD^+^-binding domains. The *Lu*HNL_lig_free and Hl_ADH superimposed with an RMSD of 8.40 Å at 669 Cα atoms, reflecting the similarity of structures to some extent. However, the difference in activity indicates the existence of very significant structural changes.

### Absence of ADH activity in *Lu*HNL and vice versa

**Figure 9:**
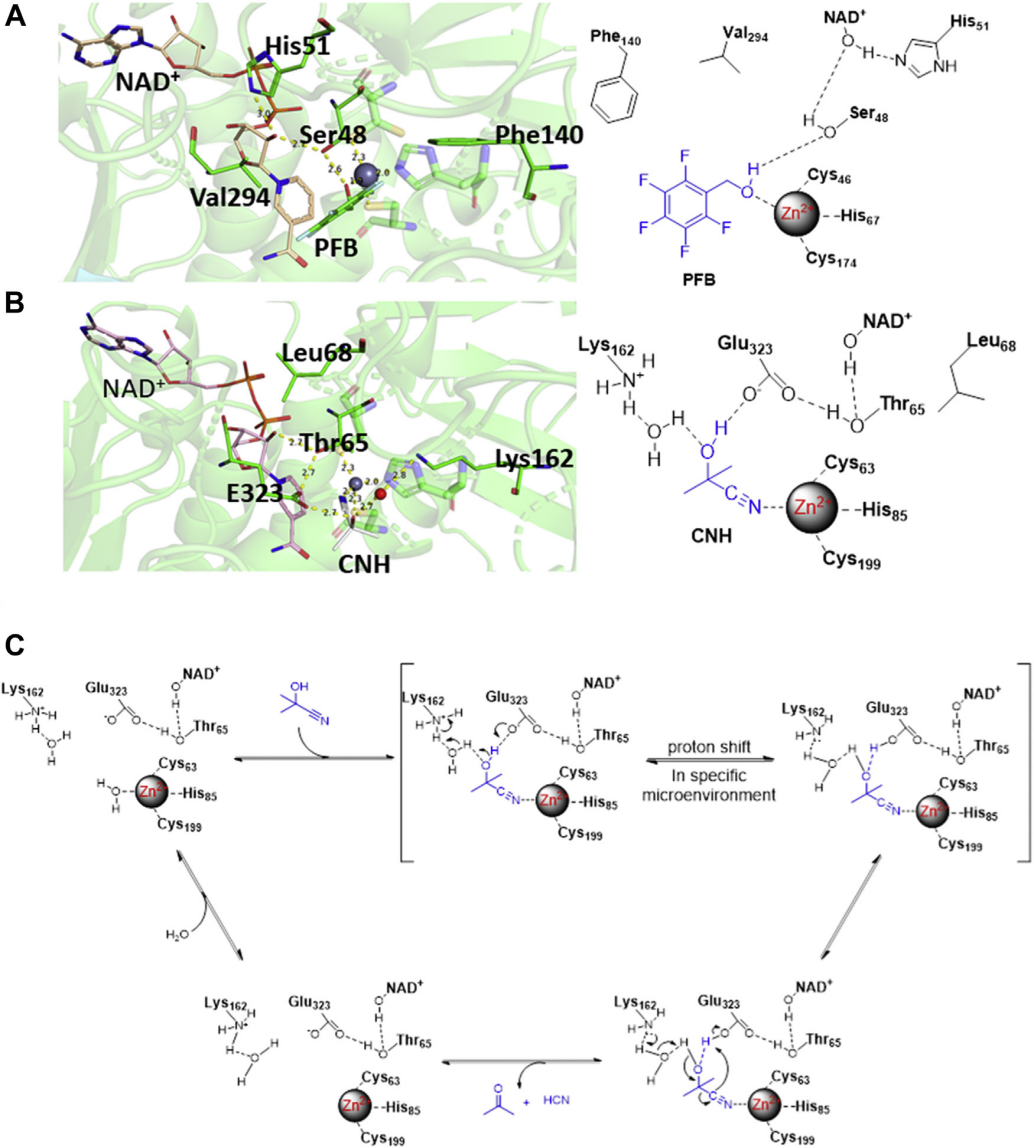
**Substrate binding patterns and proposed reaction mechanism.***A*, substrate binding pattern in Hl_ADH; (*B*) substrate binding pattern in *Lu*HNL; (*C*) proposed reaction mechanism for *Lu*HNL. CNH refers to acetone cyanohydrin, and PFB refers to 2,3,4,5,6-pentafluorobenzyl alcohol. The protein structures were displayed using PyMOL (79).

In Hl_ADH, the hydroxyl group of the substrate was bonded to the catalytic Zn^2+^ that coordinated with Cys46, His67, and Cys174 (Fig. 9*A*). The deprotonation of the hydroxyl group is carried out by a general base of His51 via a hydrogen bonding relay consisting of Ser48 and O2D of NAD^+^. The following hydride transfer from the α-carbon atom of the substrate to C4N of NAD^+^ occurs at a distance of 3.3 to 3.5 Å via substrate mobility (PDB IDs: 4NFH and 1MG0) (60, 65). However, in *Lu*HNL, it is the nitrogen atom of the substrate nitrile group that coordinates with catalytic Zn^2+^, rather than the oxygen atom of the substrate hydroxyl group. His51, which acts as a general base for deprotonation in Hl_ADH, is replaced by Leu68 in *Lu*HNL. In addition, the residues of Phe140 and Val294 in Hl_ADH were replaced by Lys162 and Glu323 in *Lu*HNL, which act as catalytic sites in the decomposition of cyanohydrin in *Lu*HNL (Fig. 9*B*). The distance of cyanohydrin alfa-C atom to C4N of NAD^+^ is about 5.1 to 5.4 Å, much further than that in Hl_ADH, suggesting that the C4N of NAD^+^ in *Lu*HNL is not directly involved in the reaction mechanism. In addition, the binding geometry of glycerol, a pseudosubstrate that bound in the catalytic pocket of ligand-free *Lu*HNL structure (*Lu*HNL-lig-free) suggests that a hydroxyl group cannot replace the water molecule to bond with catalytic Zn^2+^, even in the absence of a nitrile group (Fig. 5*B*). This may explain the slight inhibitory effect of EDTA on the activity of *Lu*HNL. The two different substrate binding patterns provide insight into differences in the activities of *Lu*HNL and Hl_ADH.

### Catalytic mechanism of *Lu*HNL

In a common catalytic mechanism of hydroxynitrile lyases, one residue acts as a base to extract the proton from the hydroxyl group, and the left cyanide anion is stabilized in a microenvironment with a positive electrostatic potential (66). Based on the complex structure of *Lu*HNL and site-directed mutagenesis results, the reaction presumably occurred in the catalytic Zn^2+^ pocket. When the cyanohydrins were bound to the catalytic sites, the hydrogen bonding relay may be perturbed in a specific microenvironment to form deprotonated Lys162 and protonated Glu323. Then the deprotonated Lys162 acts as a base to extract the proton from the hydroxyl group *via* one molecule of water, and the electrostatic interaction between positively charged Zn^2+^ and cyanide ion renders the cyanide a better leaving group. Then, the proton in the protonated Glu323 is transferred to the cyanide anion to release HCN (Fig. 9*C*). Unlike the utilization of histidine as a general base to extract the proton in the first step, as observed in *Pa*HNL (14), *Hb*HNL (32), *At*HNL (20), and *Pe*HNL (39), Lys162 acts as the base for proton extraction in *Lu*HNL. However, lysinium has much higher p*K*a value than histidinium, let alone glutamic acid. How the enzyme stabilizes the transition state consisting of deprotonated lysinium (Lys162) and protonated glutamate (Glu323) especially in a weak acidic solution is a fascinating issue. Such proton transfer from lysinium to aspartate followed with proton extraction from substrate by the deprotonated lysine has been reported in *Chua*HNL. The deprotonated lysine in *Chua*HNL was proposed to be stabilized by the desolvation effect in the hydrophobic active site (41). The desolvation effect by placing the side chain into a specific microenvironment can significantly perturb the *pK*a of a nucleophile, as detected for the buried lysine (67) and glutamic acid (68) in the interior of proteins. In *Lu*HNL, the catalytic sites (Lys162, Glu323) are located in the upper part of the catalytic pocket as shown in Figure 6*A*. When the substrate is bound to the enzyme, the substrate entry tunnel 1 is blocked by the substrate molecule. The catalytic sites are partially removed from the bulky water by shielding the substrate entry tunnel 2 in a dimer form bearing only one molecule of water for proton delivery, which may result in the p*K*a shift of Lys162 and Glu323.

### The catalytic pocket in *Lu*HNL

The complete inactivation of *Lu*HNL by site-directed mutagenesis of the catalytic residues of Thr65, Lys162, Glu323, Cys63, His85, and Cys199 supports the above proposed reaction mechanism. As discussed in the catalytic mechanism of *Lu*HNL, a hydrophobic microenvironment may be essential to stabilize the deprotonated Lys162 and protonated Glu323, which involves the tightly bound NAD^+^ and the shielded substrate entry tunnel 2 in the dimer form of *Lu*HNL (Fig. 6*A*).

In enzymatic dehydrogenation reactions, a free NAD(P)H or NAD(P)^+^ is essential to catalyze the redox reaction by providing or accepting a hydride ion. An exception is formaldehyde dehydrogenase derived from *Pseudomonas putida* (PFDH), which catalyzes the disproportionation of aldehydes without the external addition of cofactor. The structural origin is a long insertion loop in PFDH, shielding the adenine part of the bound NAD^+^ molecule from the solvent (69). However, the reaction catalyzed by *Lu*HNL is not a disproportionation reaction, so the tightly rather than covalently bound cofactor suggests that NAD^+^ is unlikely to play a redox role in *Lu*HNL. However, the loss of activity in the correctly folded apo-*Lu*HNL-R249G/S268A/E269L (*Lu*HNL-S4 in Fig. S2, Supporting information, Section 4) indicates the importance of NAD^+^ in *Lu*HNL activity. Once the *Lu*HNL loses NAD^+^, the activity will also be lost and can no longer be recovered. Presumably, instead of acting as a redox role to participate in the reaction, the NAD^+^ in *Lu*HNL is part of the catalytic pocket to define a specific microenvironment. The similar function of cofactor for an enzyme was reported in the FAD-containing *Pa*HNL, in which the oxidized cofactor of FAD is solely required for electrostatic reason, rather than a redox role (14).

In addition, it is noteworthy that, in Hl_ADH, the substrate entry tunnel is located beside the interface of two subunits, same position as the substrate entry tunnel 2 observed in *Lu*HNL, but the tunnel in Hl_ADH is still open when the substrate is bound to catalytic sites in a dimer structure, as observed from the ligand-complexed structure (PDB: 4NFH) (60) (Fig. 6*C*). However, in *Lu*HNL, the substrate entry tunnel 2 was completely shielded by the helix-α10 of another chain when two subunits assemble into a dimer. The residue Phe340 on the helix-α10, acting as a hydrophobic lid, completely covered the entire entry tunnel 2, resulting in the formation of a specific microenvironment (Fig. 6*B*). The significant decrease on the activity of *Lu*HNL by site-directed mutagenesis of Phe340 and Met74 to alanine supports this viewpoint that a semi-closed catalytic pocket is indispensable for enzyme activity.

Obviously, compared with the long and winding substrate entry tunnel 1, substrate entry tunnel 2 is a more suitable channel for substrate to enter the enzyme if it is not shielded by the residue Phe340 (Fig. 6*A*). Thus, in the process of substrate binding and product release, whether there is conformational change of two subunits in one dimer is an interesting issue. Unfortunately, in the ligand-free structure of *Lu*HNL, a pseudosubstrate of glycerol was trapped in the catalytic pocket. Thus, whether the conformation of enzyme varies with the absence or presence of a compound in the catalytic pocket remains to be studied.

### The function of Zn^2+^ in *Lu*HNL

The presence of two Zn^2+^ ions in each subunit of *Lu*HNL was confirmed by quantitative measurement. One of them that is located in the catalytic pocket is presumably involved in the catalysis of cyanohydrins decomposition via the nitrile-Zn^2+^ complex. The positively charged Zn^2+^ is responsible for the stabilization of the cyanide anion. Another Zn^2+^ complex with four cysteine residues is structurally important for the correct folding of protein. The inactivation of the enzyme by mutating the Cys121 to alanine strengthens this viewpoint.

### The Mg^2+^ ion and the S-nitrosylation of Cys265 on the surface of *Lu*HNL

Magnesium is the most abundant divalent cation in cells with diverse biochemical functions (70, 71). Statistical studies of the inner-sphere binding mode of Mg^2+^ revealed that approximately 77% of all Mg−X bonds are Mg−O bonding situations in which either water or negatively charged oxygen functionalities such as carboxylates (Asp, Glu) are the preferred ligands (72). Moreover, the distances between the cation and the oxygen atom of proteins and small molecules, as determined by crystal structure studies, vary from 2.05 to 2.25 Å, much more constrained than hexa-coordinated Ca^2+^ (70). The magnesium complex in *Lu*HNL that coordinated with Glu52, Glu140, and four molecules of waters in a distance of 2.0 to 2.2 Å is consistent with these descriptions. However, the low content of Mg^2+^ (approximately 0.35 per subunit) and active mutant of E140A suggest that it is not essential for *Lu*HNL activity. To some extent it cannot be ruled out that the Mg^2+^ was bound on the surface coincidently, because of the availability of a suitable location. In addition, the S-nitrosylation of cysteine has emerged as an important mechanism for dynamic, posttranslational regulation of most or all main classes of proteins (73). However, the failed detection of S-nitrosylation in *Lu*HNL solution ruled out that the Cys265 was modified during enzyme expression in *E. coli*. The formation of S-nitrosylation may occur in the crystallization step or diffraction experiment when exposed to high-energy X-ray. The negligible effect of C265A on enzyme activity strengthens this viewpoint.

In summary, this study elucidates the reaction mechanism of *Lu*HNL on cyanohydrin degradation and provides insights into differences in activities of *Lu*HNL and ADH, which has long been a challenge. This understanding of the novel reaction mechanism will contribute to the study of hydroxynitrile lyases and provide a new model for designing enzymes.

## Experimental procedures

### Overexpression of *Lu*HNL and purification

The gene for *Lu*HNL from *L. usitatissimum* (GenBank accession number AF024588.1) (47) was cloned into pET-15b vector (Novagen) with *NdeI* (CATATG) and *BamHI* (GGATCC) restriction sites, and a His-tag peptide and a thrombin recognition sequence (MGSSHHHHHHSSGLVPRGSHM) were attached to the N terminus. The resulting plasmid was transformed into competent JM109 *E. coli* cells, and the copied plasmid was extracted using the Gene elute Plasmid Miniprep Kit (Sigma-Aldrich). The extracted recombinant plasmid sequence was confirmed by Genetic Analyzer 3500 (ThermoFisher Scientific) using T7 promoter primer (5′-TAATACGACTCACTATAGGG-3′) and T7 terminator primer (5′-ATGCTAGTTATTGCTCAGCGG-3′). Then the recombinant plasmid was transformed into SHuffle T7 Express Competent *E. coli* (New England Biolabs) for expression. Noteworthy, there are two *Lu*HNL sequences deposited in Genbank (Y09084.1 (47) and AF024588.1 (45)), but six base variants exist in these two sequences. Five of them are nonsense mutation; the remaining one shows difference at the codon that encodes the 117th amino acid. The corresponding amino acid sequence ID of the gene sequence of AF024588.1 is AAB81956.1, which is Thr117 as we uploaded in the deposition. The corresponding amino acid sequence ID of the gene sequence of Y09084.1 is P93243.1, which is Val117 as described in the validation report. Their sequence alignments are shown in Fig. S6 (Supporting information, Section 8).

A single colony of SHuffle T7 *E. coli* harboring the plasmid of pET-15b-*Lu*HNL was inoculated into 5 ml lysogeny broth (LB) medium containing 100 μg/ml ampicillin (Amp) and cultivated at 30 °C, 300 RPM overnight. Then, 3 ml preculture was transferred into 500 ml LB medium containing 100 μg/ml Amp and cultivated at 30 °C, 150 RPM for 5 h (*A*_600_ = 0.64), then 1 mM isopropyl β-D-1-thiogalactopyranoside (IPTG) was added to induce the protein expression, and the cells were continued to cultivate at 16 °C, 120 RPM for 24 h. The cells from 4 L of medium (500 ml × 8) were harvested by centrifugation (6000*g*, 10 min, 4 °C) and the pellet was resuspended in 100 ml lysis buffer (20 mM potassium phosphate buffer [KPB], 20 mM imidazole, 500 mM NaCl, pH 7.4). The cells were disrupted by sonication in an ice bath for 30 min. Then, the debris and insoluble protein were removed by centrifugation (15,000*g*, 30 min, 4 °C). The supernatant was loaded onto a 15-ml Ni Sepharose 6 Fast flow column (GE Healthcare) equilibrated with lysis buffer (10 column volume [CV]), followed by washing with lysis buffer (10 CV). A gradient elution program was performed using 15 CV of lysis buffer (buffer A) and 15 CV of buffer B (20 mM KPB, 500 mM imidazole, 500 mM NaCl, pH 7.4) to elute the bound protein. The fractions were collected in volumes of 10 ml per tube. The active fractions were pooled and dialyzed against 20 mM KPB (pH 7.4, 5 L × 2, 4 °C). Subsequently, the active fraction was concentrated and applied to Mono Q 5/50 GL column (bed volume: 1 ml; GE Healthcare) for further purification. The bound protein was eluted with a linear gradient of 0 to 0.15 M NaCl (40 CV), 0.15 to 0.25 M NaCl (20 CV), 0.25 to 0.5 M NaCl (10 CV) in 20 mM KPB (pH 7.4). The purity of active fractions was analyzed by SDS-PAGE, and pure fractions were pooled and concentrated to 10.5 mg/ml (measured by BCA method, Takara) for further crystallization.

### Crystallization

The crystals of N-His-*Lu*HNL were prepared using the vapor diffusion sitting drop method at 20 °C in 96-well Intelli-Plates (Art Robbins Instruments). The sitting drop was prepared by mixing 1 μl of N-His-*Lu*HNL (10.5 mg/ml) with 1 μl crystallization buffer (0.1 M BIS-TRIS, pH 6.5, 20% w/v polyethylene glycol monomethyl ether 5,000, HAMPTON RESEARCH, Index, Reagent 46) (HAMPTON RESEARCH). A total of 50 μl of crystallization buffer was used as reservoir solution.

### Data collection, processing, model building, and refinement

Before subjecting the crystals to flash-freezing for X-ray diffraction, the sample for ligand-free structure of *Lu*HNL determination was prepared by soaking the crystal in solution I (crystallization buffer containing 15% (v/v) glycerol) and cryoprotectant solution II (crystallization buffer containing 25% (v/v) glycerol) successively. Similarly, the sample for *Lu*HNL–acetone cyanohydrin complex structure determination was performed by soaking the crystal in cryoprotectant solution I and cryoprotectant solution III (cryoprotectant solution II/acetone cyanohydrin: 90/10 (v/v)) for 20 min. The sample for *Lu*HNL-2-butanone cyanohydrin complex structure determination was similarly prepared by soaking the crystal in cryoprotectant solution I and cryoprotectant solution IV (cryoprotectant solution II/(*rac*)-2-butanone cyanohydrin race: 98/2 (v/v)) for 5 min.

The X-ray diffraction data of ligand-free *Lu*HNL and *Lu*HNL_CNH were collected at 100 K at the beamline BL-5A of KEK-PF with a reflection record of 0.2° per image. Another data set of *Lu*HNL soaked by (*rac*)-2-butanone cyanohydrin was collected using an in-house X-ray generator and an imaging plate (MicroMax-007HF and R-AXIS VII, Rigaku) with a reflection record of 0.5° per image. All datasets were integrated using iMosflm (74) and scaled using SCALA (75). The initial model for molecular replacement was built using the automatic molecular replacement pipeline program *BALBES* (49). All models were corrected using COOT (76) and refined using *REFMAC5* (51) and *Phenix* (52). *R*_free_ values were computed from 5% of the randomly chosen reflections that were not used for refinement. Water molecules were inserted automatically and manually into the potential electron density map. The validation of the water molecules was automatically performed according to the geometric criteria and their refined *B*-factors (B < 60 Å^2^).

### Site-directed mutagenesis of *Lu*HNL

The *Lu*HNL mutants were prepared via site-directed mutagenesis using the PrimeSTAR Mutagenesis Basal kit (Takara) with forward and reverse primers of a 27-mer oligonucleotide designed as indicated by the kit manual. The PCR was performed for 30 cycles: (denaturation 98 °C/10 s, annealing 55 °C/15 s, elongation 72 °C/40 s). The amplified PCR product was purified using a Wizard SV gel and PCR clean-up system (Promega). The resulting PCR product was transformed into JM109 *E. coli* competent cell. The recombinant plasmids were extracted from the JM109 *E. coli* and sequenced by Genetic Analyzer 3500 (ThermoFisher Scientific) using T7 promoter primer (5′-TAATACGACTCACTATAGGG-3′) and T7 terminator primer (5′-ATGCTAGTTATTGCTCAGCGG-3′). The confirmed plasmids were transformed into SHuffle T7 Express Competent *E. coli* (New England Biolabs) for expression. The purification of the mutants was performed as the protocol described for wild type.

### *Lu*HNL activity measurement

The acetone cyanohydrin degradation activity of *Lu*HNL was determined by monitoring the formation of CN^−^ ion (77). The reaction mixture was composed of an appropriate amount of enzyme, 10 mM acetone cyanohydrin (100 μl of 100 mM acetone cyanohydrin prepared in 0.1 M citric acid solution), and 400 mM citrate buffer (pH 4.5) in a total volume of 1 ml, which was monitored at room temperature by cyanide detection. For cyanide detection, 1 μl enzymatic reaction mixture was added to 199 μl oxidants solution (27 mM succinimide and 2 mM *N*-chlorosuccinimide in DIW), followed by the addition of 50 μl coupling reagent (0.2 M barbituric acid and 24% pyridine (V/V) in deionized water). The resulting mixture was incubated at room temperature for 10 min, then measured at 580 nm. One unit enzyme activity was defined as the enzyme amount needed to catalyze the formation of 1 μmol CN^−^ in 1 min.

## Data availability

The structures of *Lu*HNLs described in this paper have been deposited into Protein Data Bank. The PDB IDs for the three structures are 7VB3 for *Lu*HNL-lig-free, 7VB5 for *Lu*HNL-CNH, and 7VB6 for *Lu*HNL-BCN.

## Supporting information

This article contains supporting information (77, 78).

## Conflict of interest

The authors declare that they have no conflicts of interest with the contents of this article.
