# Supplementary material for: Structural characterization of Linum usitatissimum hydroxynitrile lyase: A new cyanohydrin decomposition mechanism involving a cyano-zinc complex
Source: J Biol Chem. 2022 Jan 29;298(3):101650. doi: 10.1016/j.jbc.2022.101650 (PMC8892092; doi:10.1016/j.jbc.2022.101650)
Supplement: Supplemental Figures S1–S6, Tables S1 and S2 [file mmc1.docx]

**Supporting Information**

**Structural characterization of *Linum usitatissimum* hydroxynitrile lyase: a new cyanohydrin decomposition mechanism involving a cyano-zinc complex**

**Daijun Zheng^[a]^, Makoto Nakabayashi^[b]^ and Yasuhisa Asano^[a]^***

^[a]^ Biotechnology Research Center and Department of Biotechnology,Toyama Prefectural University, 5180 Kurokawa, Imizu, Toyama 939-0398 (Japan), E-mail: asano@pu-toyama.ac.jp.

^[b]^ Faculty of Pharmacy, Osaka Ohtani University, 3-11-1, Nishikiori-kita, Tondabayashi, Osaka 584-8540, Japan

.

Correspondence and requests for materials should be addressed to Y. A. ([asano@pu-toyama.ac.jp](mailto:asano@pu-toyama.ac.jp)) (Tel: +81-766-56-7500)

**Table of Contents**

1. Statistics for data collection and refinement …………………………..……………………….…….......…3
2. The quantitative measurement of metal ions in *Lu*HNL ……. …….................................…….…....……....4
3. Quantitative measurement of NAD^+^ in *Lu*HNL ………………………………..…………………….….….5
4. Quantitative analysis of *Lu*HNL-R249G/S268A/E269L …………………………………………….……..….6
5. Time course for activity assay of *Lu*HNL mutants ……………………………………………………………7
6. Kinetic parameters asssy of *Lu*HNLs …………………………….…………………….……………………..8
7. EDTA effect on *Lu*HNL activity ………………………………………………………...……………………9
8. Alignment of *Lu*HNL nucleotide sequence and corresponding amino acid sequence from different deposition in GenBank……………………………………….…..………………………………………..10
9. References………………………………………………………………………………………...………..10

**1.** **Statistics for data collection and refinement**

**Table S1:** **Statistics for data collection and refinement of *Lu*HNL structures**

^[a]^ *R*_merge_ = Σ|(*I_hkl_* - <*I_hkl_*>)| / (Σ*I_hkl_*), where <*I_hkl_*> is the mean intensity of all reflections equivalent to reflection *hkl*.

^[b]^ *R*_work_ (*R*_free_) = Σ||*F*_obs_|-|*F*_calc_|| / Σ|*F*_obs_|, where 5 % of randomly selected data were used for *R*_free_.

**2. The quantitative measurement of metal ions in *Lu*HNL**

To 10 mL of *Lu*HNL enzyme solution with different concentrations (8.6 nM-610 nM) was added by 50 µL nitric acid solution (V_acid_/V_H2O_=1/2). The resulted solution was applied to Inductively Coupled Plasma Mass Spectrometry (ICP-MS, Agilent 7700, CA, USA) analysis.

**Table S2:** **Quantitative measurement of metal ions in *Lu*HNL using ICP-MS^[a]^.**


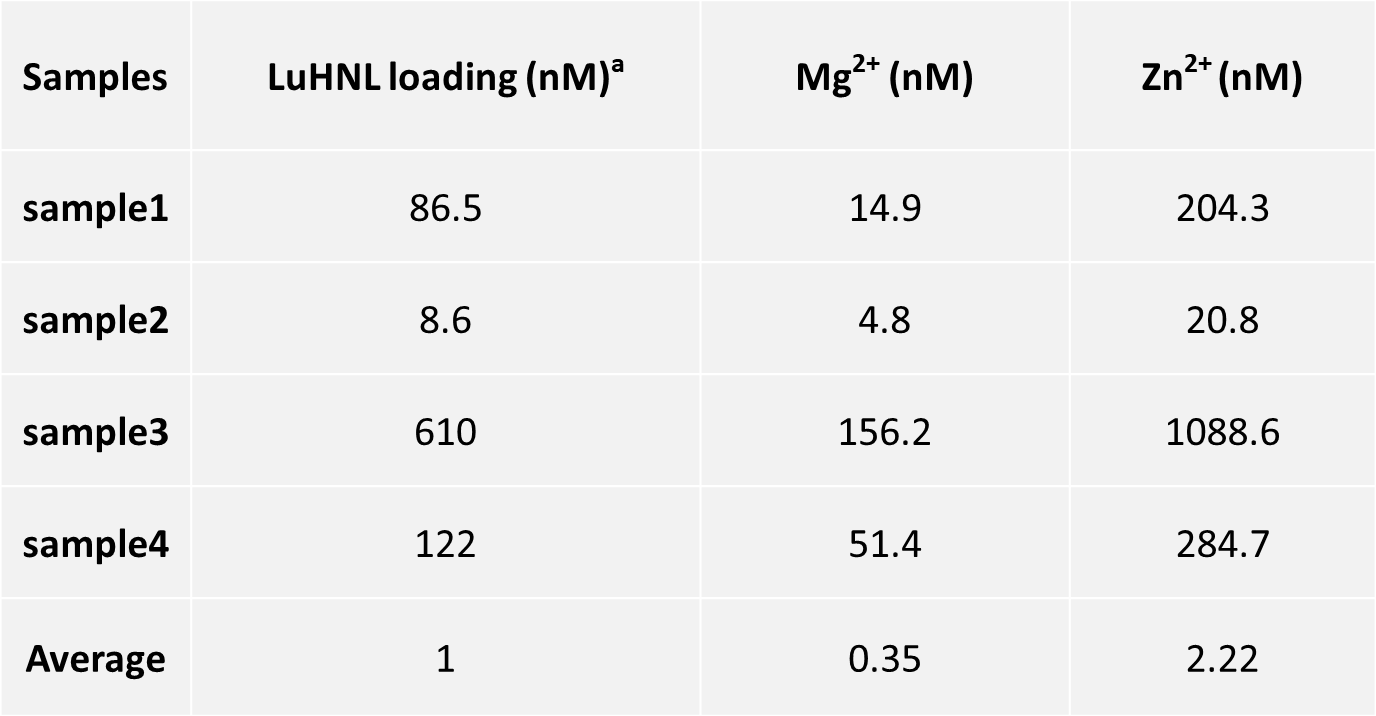


^[a]^ The concentration of *Lu*HNL was measured by BCA method (Takara, Otsu, Japan) and the *Lu*HNL molecular mass of 48 kDa was used for calculation.

**3. Quantitative measurement of NAD^+^ in *Lu*HNL**

5 mg/mL *Lu*HNL (measured by BCA method, Takara, Otsu, Japan) was incubated with 4 M guanidine in ice-bath for 1 h. The denatured *Lu*HNL and NAD^+^ cofactor were separated by filtration (12, 000×g, 20 min, 4 °C, Amicon Ultra 10K, Merck Millipore, MA, USA). The NAD^+^ in the filtrate was measured at wavelength of 260 nm and quantitatively calculated using a calibration curve prepared from standard NAD^+^ compound (Fujifilm Wako, Osaka Japan).

**Fig. S1.** **Quantitative measurement of NAD^+^ in *Lu*HNL.** (A) The UV scanning spectrum of 4 M guanidine; (B) The UV scanning spectrum of mixture of 5 mg/mL BSA and 0.1 mM NAD^+^; (C) The UV scanning spectrum of mixture of 5 mg/mL *Lu*HNL and 4 M guanidine; (D) The UV scanning spectrum of mixture of 5 mg/mL *Lu*HNL; (E) The UV scanning spectrum of 0.1 mM NAD^+^; (F) The UV scanning spectrum of the filtrate of 5 mg/mL BSA and 0.1 mM NAD^+^; (G) The UV scanning spectrum of the filtrate of 5 mg/mL *Lu*HNL and 4 M guanidine; (H) The UV scanning spectrum of the filtrate of 5 mg/mL *Lu*HNL.


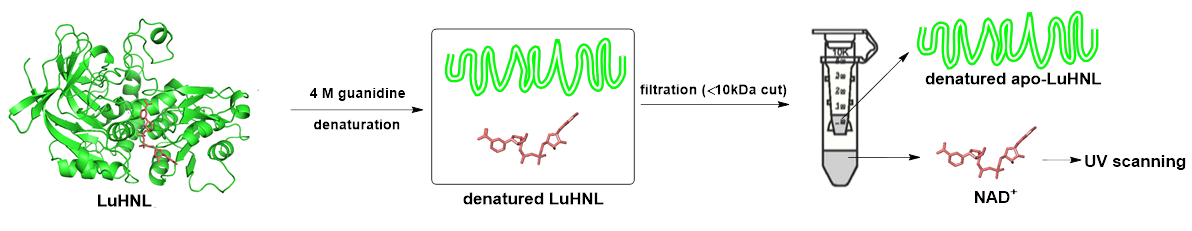


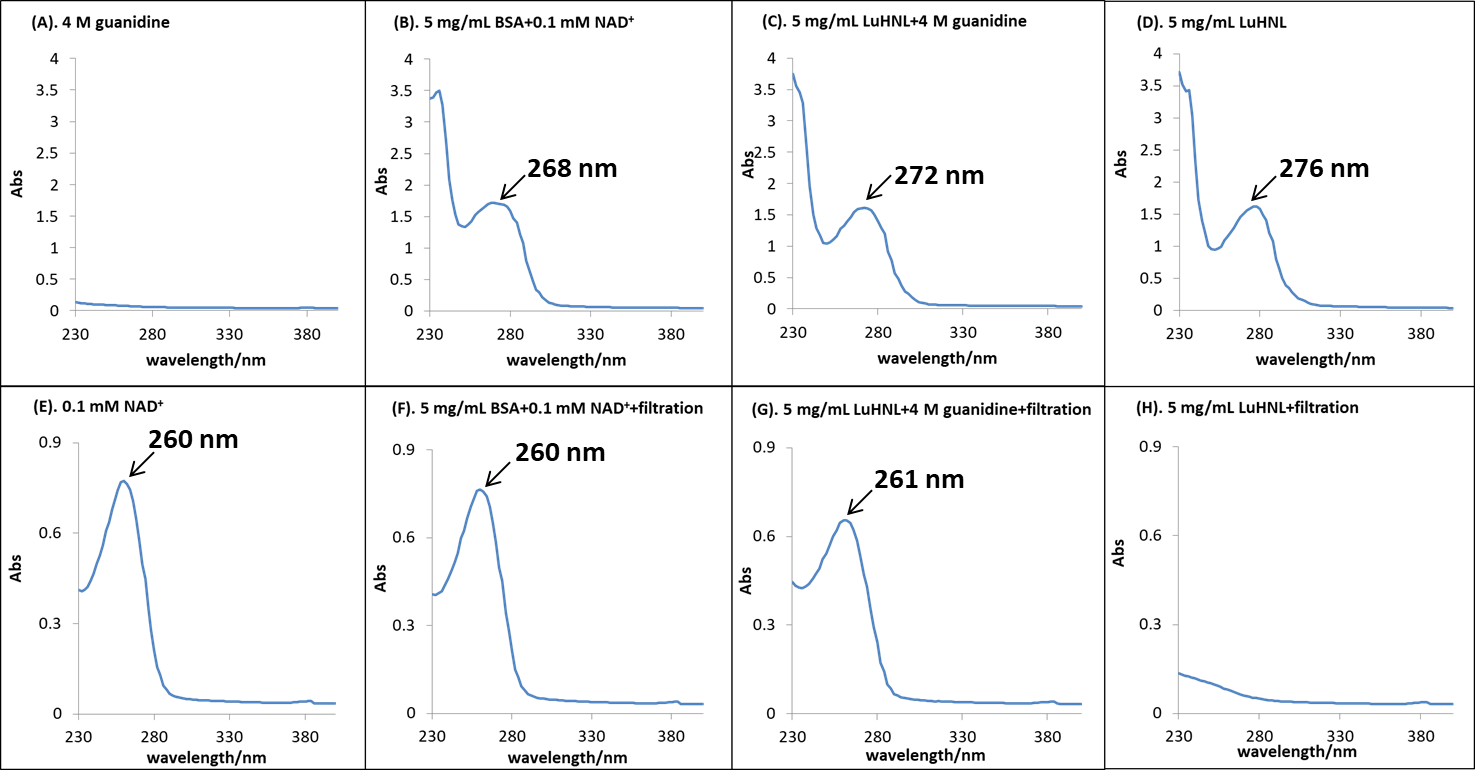


**4. Quantitative analysis of *Lu*HNL-R249G/S268A/E269L**

**
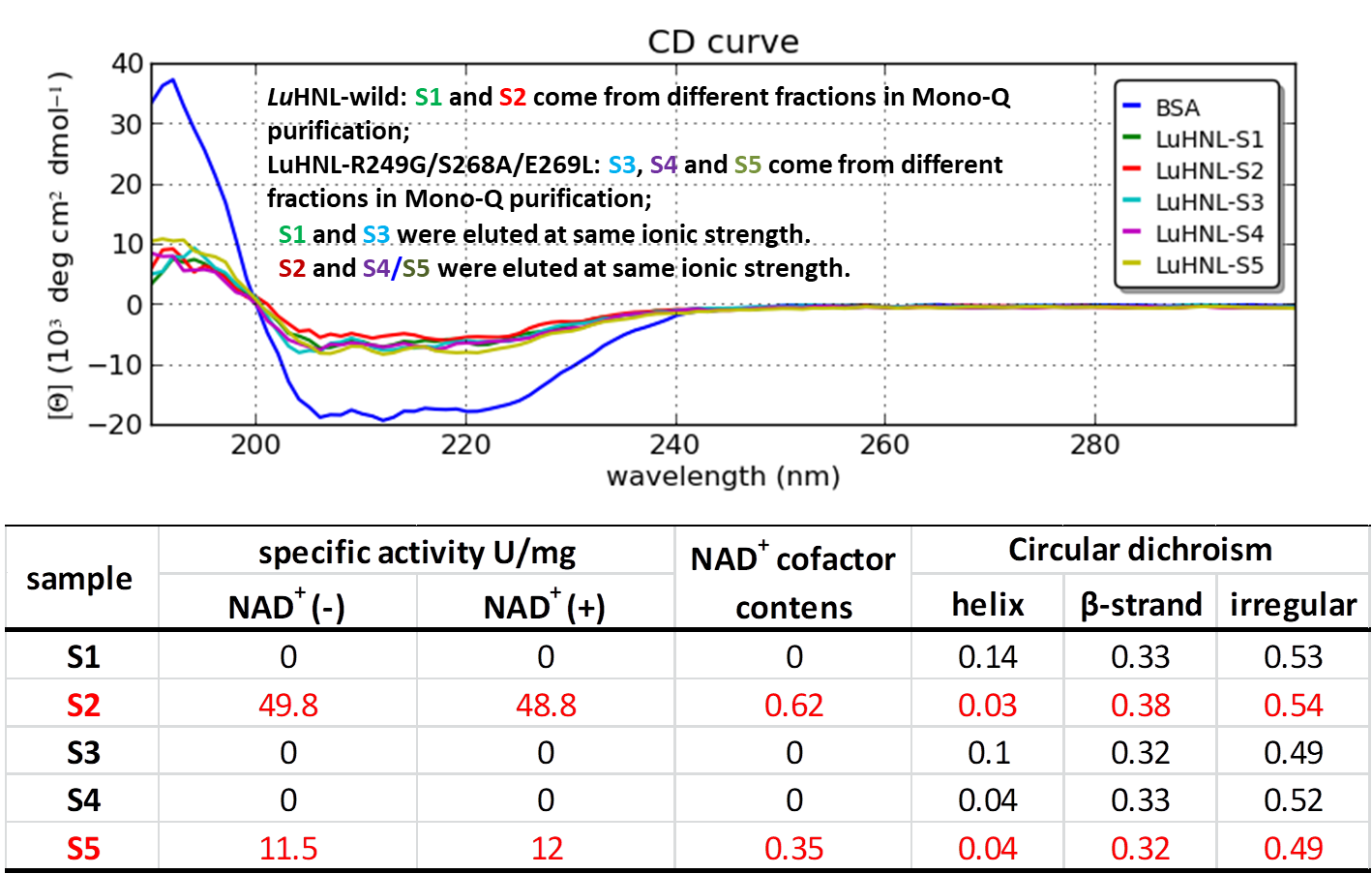
**

**Fig. S2. Quantitative analysis of *Lu*HNL-R249G/S268A/E269L.** The activity measurement with NAD^+^ was performed by addition 10 mM NAD^+^ to reaction mixture. The CD spectrum were measured with 0.05 mg/mL protein concentration (BCA method, Takara, Otsu, Japan), rt, 2 mm pathlength of cuvette. The data was processed using CAPITO program (1).

**5. Time course for activity assay of *Lu*HNLs**

**
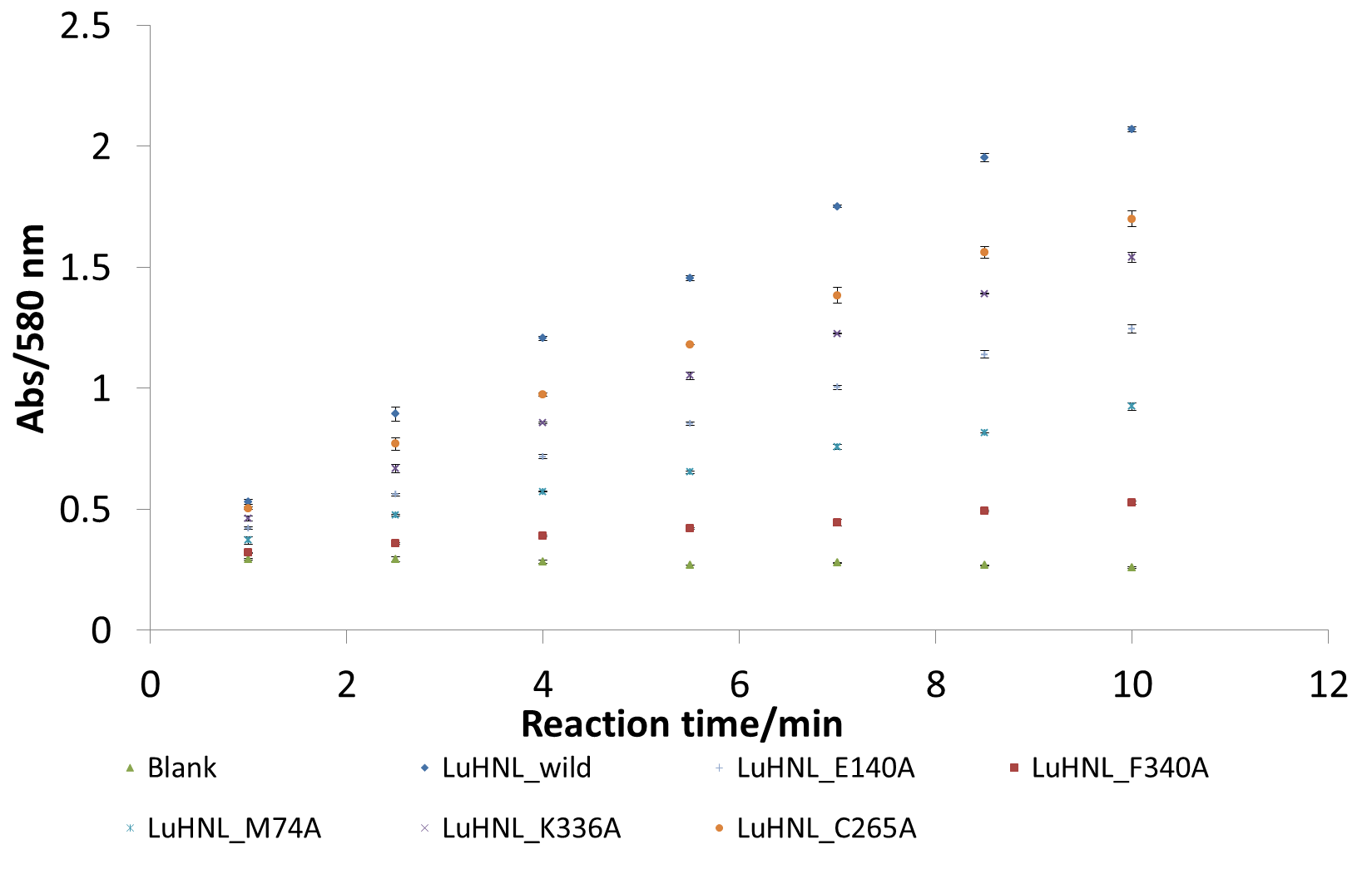
**

**Fig. S3. The time course for activity assay of *Lu*HNLs.** The acetone cyanohydrin degradation activity of *Lu*HNL was determined by monitoring the formation of CN^-^ ion (2). The activity assay for *Lu*HNL-wild, *Lu*HNL_E140A, *Lu*HNL_K336A, *Lu*HNL_C265A were performed at r.t. with 1 mL reaction mixture containing 10 µg purified enzyme, 10 mM acetone cyanohydrin, 400 mM citrate buffer (pH 4.5); The activity assay for *Lu*HNL-F340A, *Lu*HNL_M74A were performed at r.t. with 1 mL reaction mixture containing 50 µg purified enzyme, 10 mM acetone cyanohydrin, 400 mM citrate buffer (pH 4.5).

**6. Kinetic parameters assay of *Lu*HNLs**

**
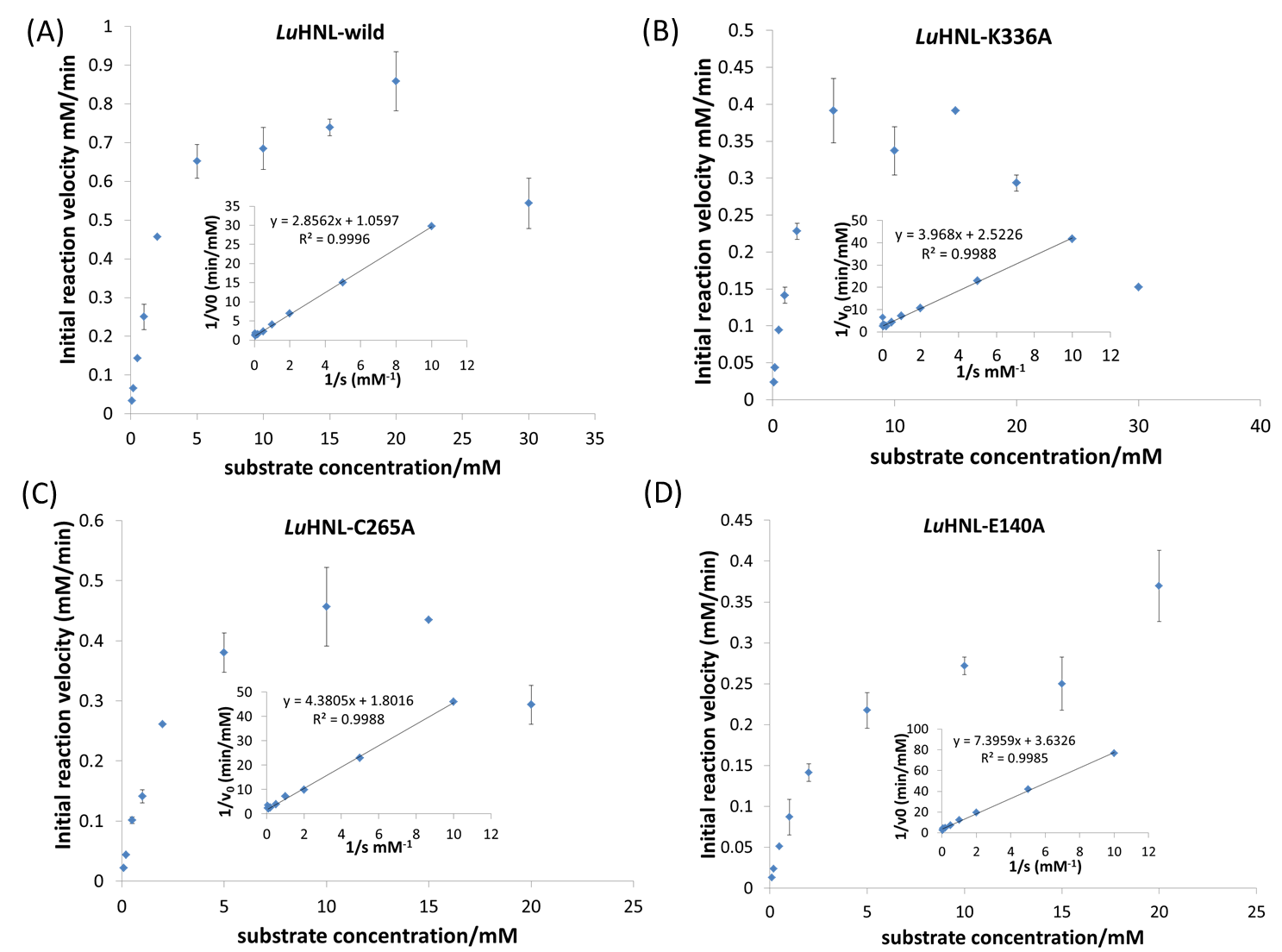
**

**
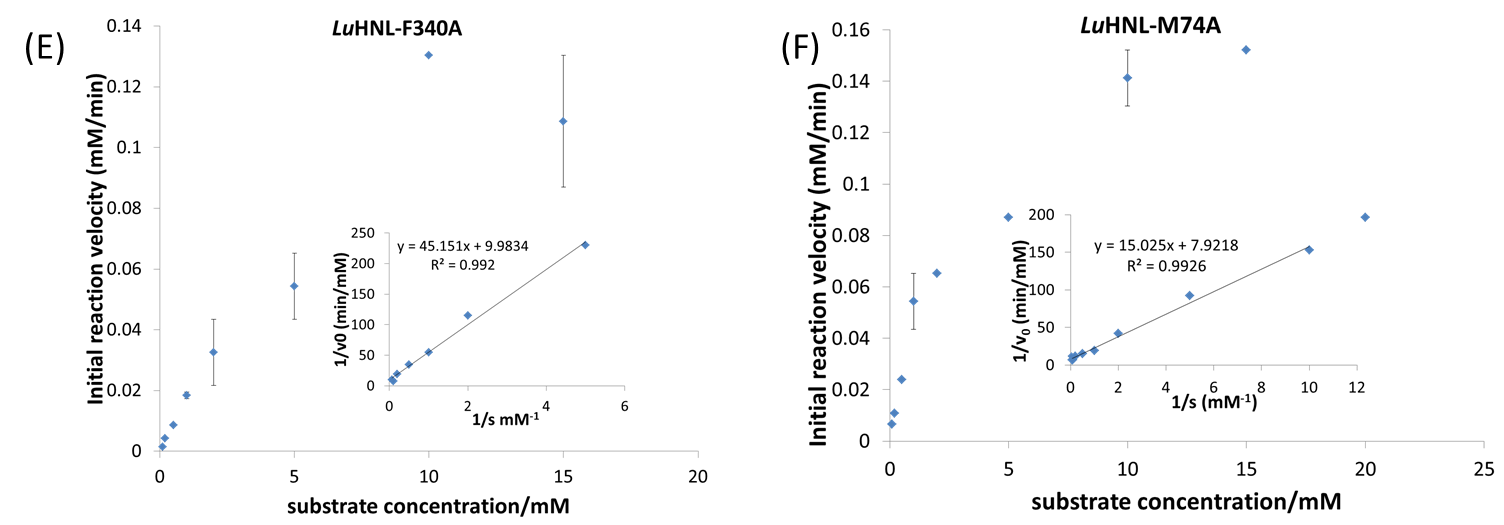
**

**Fig. S4. Kinetic parameters assay for *Lu*HNLs.** They were measured at r.t. with 200 µL reaction mixture containing 2 µg (*Lu*HNL-wild, *Lu*HNL_E140A, *Lu*HNL_K336A, *Lu*HNL_C265A) or 20 µg (*Lu*HNL_M74A, *Lu*HNL_F340A) enzyme, 400 mM citrate buffer (pH 4.5), 0.1 mM~30 mM acetone cyanohydrin. The initial reaction velocities at each substrate concentration were measured by time course.

**7. EDTA effect on *Lu*HNL activity**

**
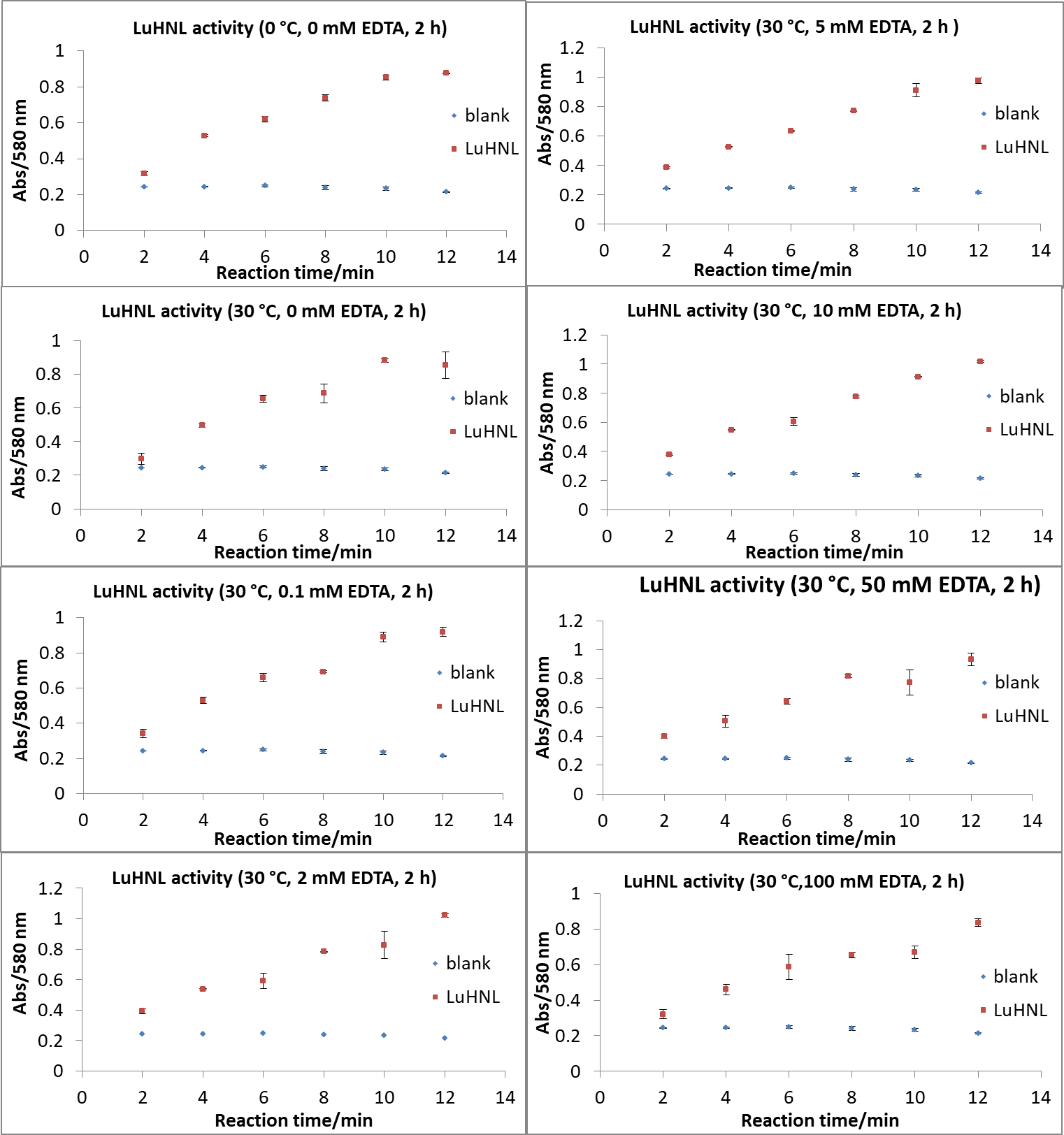
**

**Fig. S5. Investigation of EDTA effect on *Lu*HNL activity.** 400 µL of 0.28 mg/mL purified *Lu*HNL were incubated with different concentration of EDTA (0.1 mM-100 mM) at 0 °C or 30 °C for 2 h. The resulting solutions were used for activity assay directly. The activity assay were performed at r.t. with 1 mL reaction mixture containing 20 µL treated enzyme solution, 10 mM acetone cyanohydrin, 400 mM citrate buffer (pH 4.5).

**8. Alignment of *Lu*HNL nucleotide sequence and corresponding amino acid sequence from different deposition in GenBank**

**
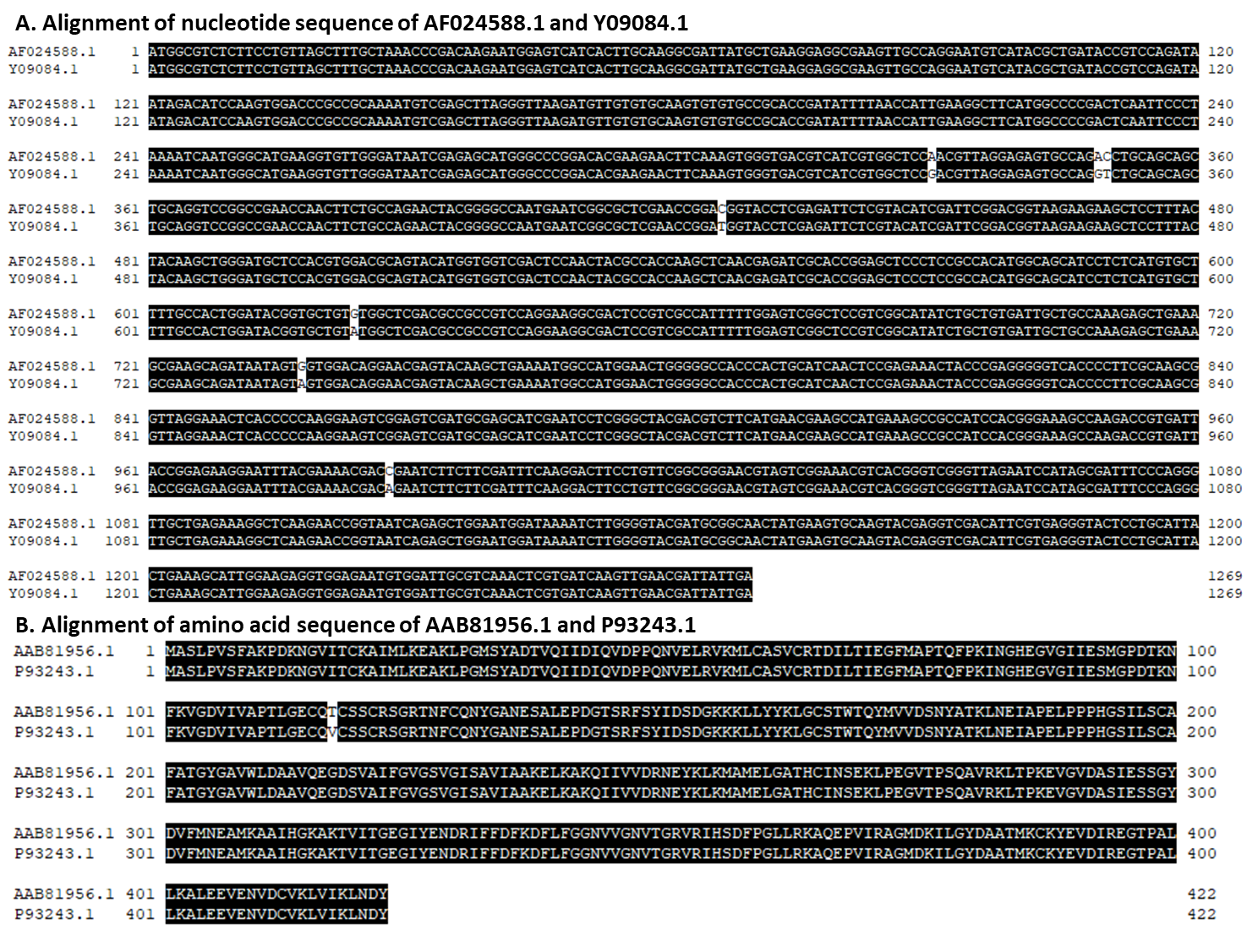
**

**Fig. S6.** Alignment of *Lu*HNL nucleotide sequences and corresponding amino acid sequences from different deposition in GenBank. The amino acid sequence of AAB81956.1 is corresponding to nucleotide sequence of AF024588.1; the amino acid sequence of P93243.1 is corresponding to nucleotide sequence of Y09084.1.

**References**

1. Wiedemann, C., Bellstedt, P., and Görlach, M. (2013) CAPITO—a web server-based analysis and plotting tool for circular dichroism data. *Bioinform.* **29**, 1750-1757

2. Lambert, J. L., Ramasamy, J., and Paukstelis, J. V. (1975) Stable reagents for the colorimetric determination of cyanide by modified Koenig reactions. *Anal. Chem.* **47**, 916-918
